# Automated detection of C-shaped canals in mandibular second molars from panoramic radiographs: comparing single and ensemble convolutional neural networks within a 2-stage pipeline

**DOI:** 10.1007/s00784-026-06868-x

**Published:** 2026-04-23

**Authors:** Yunus Emre Çakmak, Kürşat Er

**Affiliations:** Department of Endodontics, Faculty of Dentistry, Akdeniz University, Dumlupınar Bulv., 07058 Campus, Antalya, Türkiye

**Keywords:** Artificial intelligence, C-shaped canal, Convolutional neural networks, Deep learning, Object detection, Panoramic radiography

## Abstract

**Objectives:**

This study aimed to develop and validate a two-stage deep-learning workflow that automatically localizes mandibular second molars on panoramic radiographs and classifies C-shaped canal presence as an explainable, clinically deployable decision-support tool.

**Materials and methods:**

A total of 1,252 panoramic radiographs (2015–2025) were retrieved from a digital archive. Stage 1 employed YOLOv8 to detect teeth #37 and #47. Stage 2 applied three CNNs (DenseNet-169, EfficientNet-B6, ConvNeXt-Base) and ensemble approaches to classify C-shaped canals on YOLO-derived crops. Data were split 70/15/15 for training/validation/testing. Detection was evaluated using IoU and mAP; classification via accuracy, sensitivity, specificity, F1-score, and AUC. Pairwise AUC comparisons used DeLong test with Bonferroni correction. Grad-CAM provided visual explanations.

**Results:**

YOLOv8 achieved high localization performance, reliably supplying regions of interest to classifiers. ConvNeXt-Base demonstrated the most consistent discrimination with top-tier accuracy and F1-score. A binary EfficientNet-ConvNeXt ensemble performed comparably to the best individual model, while the tertiary ensemble showed no improvement. DeLong analysis revealed statistically significant AUC advantage of ConvNeXt-Base over DenseNet-169 for tooth #47 after correction.

**Conclusions:**

This two-stage pipeline enables accurate, automation-ready detection of C-shaped canals from routine panoramic radiographs, potentially informing patient triage and CBCT indication decisions. Multicenter validation is warranted before clinical deployment.

**Clinical relevance:**

This AI system offers a practical screening tool using existing panoramic radiographs to identify high-risk patients, potentially improving treatment planning efficiency, reducing unnecessary CBCT referrals, and alerting clinicians to complex anatomy before endodontic therapy.

**Supplementary Information:**

The online version contains supplementary material available at 10.1007/s00784-026-06868-x.

## Introduction

Endodontics encompasses a broad spectrum ranging from diagnostic evaluation of orofacial pain to regenerative procedures [1]. The foundation of successful endodontic treatment lies in accurate understanding and effective management of the complex anatomy of the root canal system. Anatomical variations, particularly complex structures such as C-shaped canal morphology, pose significant challenges in both diagnostic and treatment phases [2]. C-shaped canals are anatomical variations resulting from incomplete fusion of Hertwig’s epithelial root sheath on the lingual and buccal surfaces during development [2]. According to the classification system developed by Fan et al. [3, 4] this morphology can exhibit variable configurations (C1-C5) along the root length. The prevalence of C-shaped canals shows significant variations across geographic and ethnic groups, ranging from 2.7% to 52% in various Asian populations [5–7]. This high prevalence and risk of treatment complications make early detection of C-canal morphology clinically critical.

The presence of C-shaped canal systems complicates chemomechanical preparation, irrigation, and obturation due to wide fin-shaped canal spaces and thin dentin walls, increases the risk of strip perforation, and elevates treatment failure rates [2, 7, 8]. Indeed, endodontic treatment failure rates in teeth with C-canal morphology have been reported to reach up to 23.8% in narrow isthmus types [7]. Conventional 2D radiographs have serious limitations in detecting C-shaped canals due to superimposition effects and structural overlap. Studies by Cooke and Cox [9] and Lambrianidis et al. [10] demonstrated that C-canal morphology cannot be reliably identified with conventional radiography. Although cone-beam computed tomography (CBCT) is accepted as the gold standard [11, 12], it has limitations regarding radiation dose, cost, and practical applicability for every case. This situation highlights the need for alternative approaches that can reliably predict C-canal presence from routine panoramic radiographs.

In recent years, applications of artificial intelligence (AI) and deep learning technologies in dental radiology have been rapidly expanding [13–15]. Convolutional neural networks (CNNs) have demonstrated the capacity to detect complex anatomical structures from dental images with high accuracy. Pioneering studies on C-canal detection from panoramic radiographs have revealed the potential of deep learning models in this field. While Jung et al. [16] achieved 95.1% accuracy with an Xception-based model, another study [17] reached 98.4% accuracy with a lightweight CNN architecture. However, most existing models require manual tooth localization or employ single-stage classification approaches. It should be noted that the term automation in the present study refers specifically to the inference stage of the AI workflow, in which mandibular second molars are automatically localized and classified for C-shaped canal presence on panoramic radiographs without manual preprocessing. As in most supervised deep learning applications in medical imaging, expert annotation was required during dataset preparation to establish reliable ground-truth labels. In the present study, CBCT imaging served as the reference standard for determining the presence or absence of C-shaped canals, and labels were assigned by experienced endodontists. These annotations were used exclusively for model training and validation, whereas the final inference pipeline operates fully automatically on panoramic radiographs. Although the YOLO (You Only Look Once) algorithm [18] demonstrates high performance in real-time object detection, 2-stage pipeline approaches combining automatic tooth detection with C-canal classification in dental images have not been sufficiently investigated in the literature. Furthermore, the comparative performance of different advanced CNN architectures and their ensemble combinations in C-canal detection has not been systematically evaluated.

A 2-stage architecture was intentionally adopted because the target finding in this study is not the entire panoramic image but a specific tooth-level anatomical configuration within mandibular second molars. Panoramic radiographs contain extensive anatomical structures and background complexity, which may limit the effectiveness of direct end-to-end prediction from the full image. Previous studies have also highlighted the challenges of identifying C-shaped canal morphology using conventional panoramic imaging due to anatomical overlap and structural variability [10, 16]. A sequential workflow that first localizes the mandibular second molar and subsequently classifies C-shaped canal morphology enables the model to operate on anatomically focused regions of interest rather than the entire radiograph. Such modular AI pipelines are increasingly recommended in dental imaging applications because they reduce background interference, allow independent optimization of detection and classification modules, and facilitate clinical integration of decision-support systems [13].

The aim of this study is to develop a 2-stage deep learning system capable of automatically detecting C-shaped canal morphology in mandibular second molars (#37 and #47) from panoramic radiographs and to comparatively evaluate the performance of different CNN architectures. The system performs automatic localization of target teeth using YOLOv8-based object detection in the first stage, followed by C-canal classification using 3 different advanced CNN architectures (DenseNet-169, EfficientNet-B6, ConvNeXt) and their binary and tertiary ensemble combinations in the second stage. Our hypothesis is that this 2-stage approach can achieve clinically reliable C-canal detection without requiring manual intervention and that ensemble combinations of different architectures can further improve detection performance compared to single models.

## Materials and methods

### Study design and ethical approval

This retrospective, single-center diagnostic accuracy study aimed to automatically detect C-shaped canal morphology in mandibular second molars on panoramic radiographs using a 2-stage AI pipeline. The study protocol was approved by the Akdeniz University Institutional Review Board in accordance with the Declaration of Helsinki (Approval No.: TBAEK-625). Reporting adhered to the STARD-AI [19] (Standards for Reporting of Diagnostic Accuracy Studies using AI) guideline. All radiographic data were anonymized, personally identifiable information was removed, and the data were used solely for scientific purposes.

### Participants and data source

Panoramic radiographs were retrospectively collected from patients who presented to ……. University Faculty of Dentistry between January 2015 and June 2025 for routine dental examination, orthodontic evaluation, or treatment planning. Images were obtained from the faculty’s digital archive system, Metasoft Dentasist (version 4.1.168, Eskişehir, Turkey). A total of 1.252 digital panoramic radiographs were included using consecutive sampling. Patient ages ranged from 18 to 65 years; sex distribution could not be retrospectively determined due to archive system limitations. The dataset represents patients treated in a tertiary dental center and primarily reflects a Middle Eastern/Mediterranean population. Because the prevalence of C-shaped canal morphology is known to vary among different ethnic populations, this demographic context should be considered when interpreting the findings [5–7].

## Inclusion and exclusion criteria

Inclusion criteria: (a) presence of healthy, fully developed mandibular second molars (#37 and/or #47), (b) clear visualization of mandibular posterior region, (c) diagnostic quality images (adequate contrast and resolution). Exclusion criteria: (a) images with severe artifacts, blurring, or positioning errors, (b) extensive periapical lesions or advanced periodontal bone loss around teeth of interest, (c) presence of treatments that preclude assessment of root canal anatomy. Of the 1,896 images collected, 1,252 were included in the analysis; 644 were excluded due to unavailability of CBCT imaging, image artifacts, or incomplete root development. CBCT availability was required in order to establish a reliable reference standard for C-shaped canal identification. Because panoramic radiographs alone cannot reliably determine the presence of C-shaped canal morphology due to superimposition and two-dimensional projection limitations, CBCT evaluation was used to confirm the ground-truth labels. This approach ensured accurate labeling of the dataset used for supervised deep learning model training and evaluation. This approach was preferred to reflect real-world clinical diversity and objectively evaluate the model’s generalizability.

### Image characteristics and technical parameters

All panoramic radiographs were recorded in JPEG format (.jpg) at 1914 × 1024 pixel resolution and 8-bit grayscale. Images were analyzed in their original state without preprocessing or filtering. Images were obtained from four different panoramic radiography devices used at the faculty (Planmeca ProMax Dimax 4, KaVo OP 3D Pro, Carestream CS 8100SC, Morita VeraView IC5). Since information about which device produced each image was not systematically matched, no device-based stratification was performed; this contributed to the blinding principle and prevented potential device-related bias (detailed device parameters are presented in Supplementary Material Table S1).

#### Reference standard

CBCT served as the primary reference standard for determining the presence of C-shaped canals. CBCT images were acquired using Veraview X800 (J. Morita Mfg. Corp., Kyoto, Japan) at 99 kVp, 4.8 mA, and 35.8 s. Two FOV protocols were applied: 15 × 15 × 14.1 cm for jaw-focused acquisitions and 4 × 4 × 4 cm for detailed tooth-based examinations. Axial and horizontal (multiplanar) reconstructions of mandibular second molars (#37 and #47) were reviewed to establish the ground truth. In line with the study aim, the ground truth was defined as a binary per-tooth label (C-shaped canal present = 1 vs. absent = 0). CBCT validation was available for all included patients and was used solely to derive these labels and enhance label reliability; CBCT data were not used as input for model training or inference.

## Blinding procedure

Experts conducting reference standard evaluation worked completely independently of the model development process and were not informed of AI system outputs. Similarly, researchers worked blinded to individual patient information during model development and testing phases. Test dataset separation was performed by random sampling, and test set images were never used in any phase of the training process.

### Sample size determination

Post-hoc power analysis using GPower 3.1.9.7 demonstrated that with the achieved sensitivity of 96.08% and sample size of 153 C-shaped canal positive cases, the study attained > 99% statistical power (α = 0.05, two-tailed test against null hypothesis of 50% random classification). Similarly, with 216 negative cases and 97.69% specificity, power exceeded 99%, confirming adequate sample size for reliable diagnostic accuracy estimation.

## Dataset partitioning

A total of 1.252 panoramic radiographs were divided into 3 subsets using stratified random sampling: 70% training (*n* = 877), 15% validation (*n* = 187), 15% test (*n* = 188). This process was performed using a Python script (splitter.py), and 15% of all data were reserved for testing purposes. To prevent potential data leakage, dataset partitioning was performed at the panoramic radiograph level prior to ROI extraction. Consequently, all tooth crops derived from the same panoramic image were assigned to the same dataset subset (training, validation, or test). This ensured that no tooth images originating from the same radiograph or patient were distributed across different dataset partitions. Thus, the CNN models were trained and evaluated using completely independent image sets, minimizing the risk of information leakage and ensuring unbiased performance evaluation. Training and validation sets were used as the model development cohort; the test set was reserved for final performance evaluation. The determination of a 15% test set ratio was performed in accordance with the data partitioning standard commonly used in previous AI studies. Due to bilateral evaluation of mandibular second molars, 188 panoramic images contain potential evaluation of up to 376 teeth (each patient’s #37 and #47).

For CNN model training, cropped tooth regions (ROI) were extracted from the training and validation panoramic images. The resulting training dataset consisted of 799 C-shaped canal positive and 1,117 non-C-shaped canal images, totaling 1.916 cropped tooth images. It should be noted that these values represent tooth-level samples extracted from panoramic radiographs rather than patient-level prevalence. Because each panoramic radiograph may contain two mandibular second molars (#37 and #47), multiple tooth samples could be obtained from a single image and used as regions of interest for CNN training. Therefore, the reported counts reflect the number of cropped tooth regions used for model development rather than the prevalence of C-shaped canals in the patient population. No oversampling, artificial class balancing, or case enrichment strategies were applied; the dataset composition reflects the naturally occurring distribution of C-shaped and non–C-shaped canal morphologies among the CBCT-validated cases included in this study. A separate test set of 340 cropped tooth images (142 C-shaped canal positive, 198 non-C-shaped canal) was created for detailed ensemble model performance analysis. This segmented approach allowed evaluation of both the complete two-stage pipeline performance on full panoramic radiographs and isolated CNN classification accuracy on standardized tooth ROIs.

### 2-stage AI system

The developed system has a pipeline architecture containing two sequential modules: (1) YOLOv8-based automatic tooth detection (object localization), (2) convolutional neural network-based C-shaped canal classification.

## Stage 1 - automatic tooth detection (YOLOv8)

The medium version of YOLOv8 architecture (YOLOv8m) was used for object detection. The model was fine-tuned on dental radiographs starting from weights pre-trained on the Microsoft COCO dataset (transfer learning). During training, all panoramic images were standardized to 640 × 640 pixels using letterbox technique; black padding was added while preserving original aspect ratio.

Two classes were defined using LabelImg software (v1.8.6): “Tooth #37” (left mandibular second molar) and “Tooth #47” (right mandibular second molar). Bounding box coordinates (x_center, y_center, width, height) for each tooth were recorded in normalized format as.txt files suitable for YOLO format. Labeling was independently performed by 2 endodontists with at least 5 years of clinical experience; in cases of disagreement, consensus was achieved through a third expert opinion.

YOLOv8 (Ultralytics; Python 3.10, PyTorch 2.0) was fine-tuned for automatic localization of mandibular second molars. Training followed a standard regimen with early stopping, AdamW optimization, and cosine LR scheduling; automatic mixed precision was enabled. Data augmentation included mosaic (disabled in late epochs) and flips to improve robustness while preserving anatomical plausibility. Inference used conventional YOLO thresholds for confidence and Non-Maximum Suppression. Full hyperparameters and implementation details are provided in the Supplementary Methods.

### Stage 2 - C-shaped canal classification (CNN)

Relevant tooth regions (ROI) were automatically cropped using bounding box coordinates detected by YOLOv8. Cropped images were evaluated with three different CNN architectures: DenseNet-169, ConvNeXt-Base, and EfficientNet-B6. These architectures were selected due to their proven performance in medical imaging and different feature extraction strategies.

These architectures were chosen because they represent complementary deep learning design strategies that have demonstrated strong performance in medical image analysis. DenseNet-169 employs dense connectivity patterns that facilitate feature reuse and efficient gradient propagation, which can be advantageous for detecting subtle anatomical features in radiographic images. EfficientNet-B6 utilizes compound scaling to balance network depth, width, and input resolution, enabling efficient extraction of multi-scale features from dental radiographs. ConvNeXt represents a modern convolutional architecture inspired by transformer design principles and has achieved state-of-the-art performance in image classification tasks. By evaluating these complementary architectures and their ensemble combinations, the present study aimed to compare different feature extraction strategies and investigate whether ensemble learning could improve the robustness of C-shaped canal detection on panoramic radiographs.

Three CNN architectures were used: DenseNet-169 (224 × 224 pixels, ImageNet1K_V1 weights, dense connectivity with 1664→768→384→2 classifier), ConvNeXt-Base (384 × 384 pixels, GELU activation, channel attention mechanism), and EfficientNet-B6 (528 × 528 pixels, compound scaling, spatial attention). All models used dropout (0.5), batch normalization, and were fully fine-tuned on the dental dataset (detailed architectures in Supplementary Methods S5).

### Model training and validation strategy

All models were trained using 10-fold stratified cross-validation on NVIDIA A100 GPU (80 GB, Google Colab Pro+) with 100 epochs and early stopping (25-epoch patience). AdamW optimizer with model-specific learning rates (DenseNet-169/EfficientNet-B6: 3 × 10⁻⁴, ConvNeXt: 2 × 10⁻⁴), CosineAnnealingWarmRestarts scheduler (T_0 = 10), and gradient clipping (1.0) were employed. Batch sizes were optimized per model (DenseNet-169: 48, ConvNeXt: 32, EfficientNet-B6: 28) with dynamic adjustment for memory efficiency. Mixed precision training (FP16) accelerated computation (full training configuration in Supplementary Methods S5).

### Data augmentation

Comprehensive data augmentation was performed using Albumentations library, including geometric transformations (horizontal/vertical flips, rotation, shift-scale-rotate), elastic deformations (elastic transform, grid and optical distortion), pixel-level adjustments (brightness-contrast, CLAHE, HSV), noise injection (Gaussian noise/blur, motion blur), and coarse dropout. All augmentations were randomly applied during training with probabilities ranging from 5% to 80%, while validation and testing used only standard center crop and normalization.

### Ensemble strategies

Seven different configurations were evaluated to exceed single model performance:

Single models (*n* = 3): DenseNet-169, ConvNeXt-Base, EfficientNet-B6 (each independent prediction).

Binary ensemble models (*n* = 3):

- DenseNet-169 + ConvNeXt-Base (50%−50%).
- DenseNet-169 + EfficientNet-B6 (50%−50%).
- ConvNeXt-Base + EfficientNet-B6 (50%−50%).

Tertiary ensemble model (*n* = 1):

- DenseNet-169 (35%) + ConvNeXt-Base (35%) + EfficientNet-B6 (30%).

**Fig. 1:**
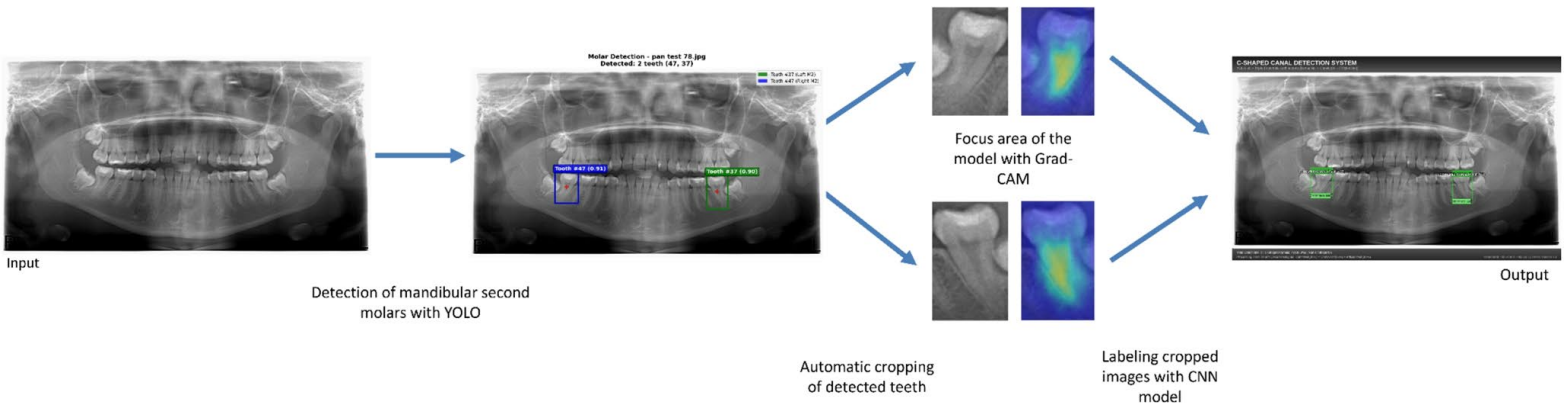
Workflow of the 2-stage C-shaped canal detection system. The YOLO model detects mandibular second molars on the panoramic radiograph and performs automatic cropping. The cropped tooth images are then passed to the ensemble CNN classifier for C-shaped canal classification. Grad-CAM analysis highlights the anatomical regions that informed the model’s decisions

Ensemble predictions were calculated by taking weighted average of each model’s softmax outputs. Test Time Augmentation (TTA) was applied by taking predictions over 5 different augmentation versions (original, horizontal flip, vertical flip, rotate 90°, rotate 270°) for each model; the average of these predictions was used as final model output. The complete workflow is schematically illustrated in Fig. 1.

### Performance Metrics

For YOLO Model:

- Intersection over Union (IoU): Overlap ratio between predicted bounding box and ground truth.
- Mean Average Precision (mAP@0.5): Average precision at IoU ≥ 0.5 threshold.
- mAP@[0.5:0.95]: Average of mAPs calculated with IoU thresholds from 0.5 to 0.95 in 0.05 increments (COCO metric).
- Precision, Recall, F1-score: Class-based detection performance.

For CNN Models:

- Accuracy: (TP + TN)/(TP + TN + FP + FN).
- Sensitivity/Recall: TP/(TP + FN).
- Specificity: TN/(TN + FP).
- Precision: TP/(TP + FP).
- F1-Score: 2 × (Precision × Recall)/(Precision + Recall).
- AUC (Area Under ROC Curve): Area under Receiver Operating Characteristic curve.

Where TP (True Positive): cases with C-canal correctly detected; TN (True Negative): cases without C-canal correctly classified; FP (False Positive): cases without C-canal incorrectly classified as positive; FN (False Negative): cases with C-canal that were missed.

### Web-based clinical interface

**Fig. 2:**
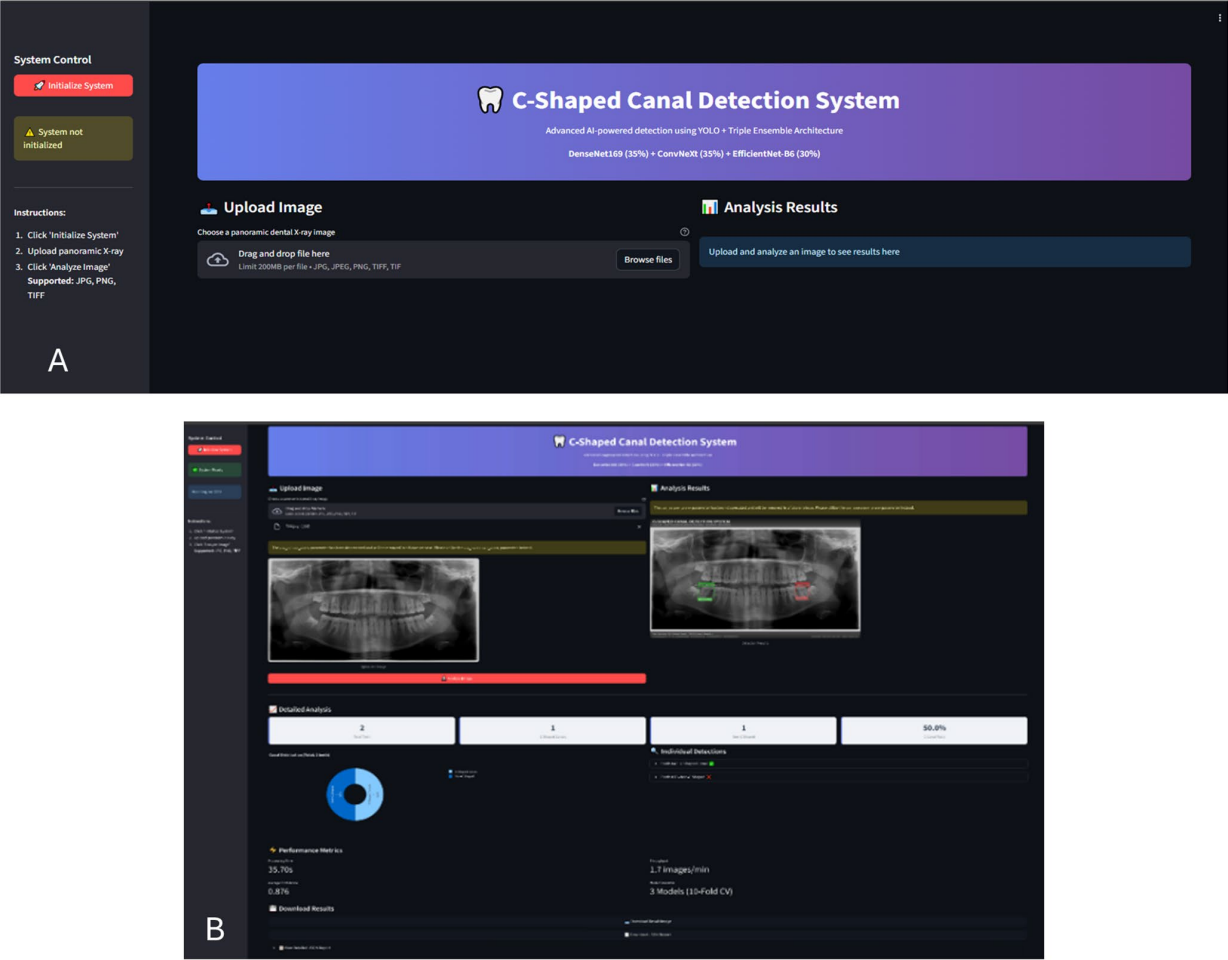
C-shaped canal detection system- Streamlit-based web interface (**A**) Home screen: a step-by-step guidance panel, a drag-and-drop uploader for panoramic images, and the “Analysis Results” display. The header summarizes the pipeline: YOLOv8 for mandibular molar detection, followed by a three-model ensemble (DenseNet-169, ConvNeXt-Base, EfficientNet-B6) for C-shaped canal classification. (**B**) Example analysis view: detected mandibular second molars are boxed on the panoramic radiograph (green = C-shaped canal positive; red = negative). Side/bottom panels present per-tooth probability/decision outputs, class counts, and performance summaries. For each detected tooth, the system extracts the ROI, computes ensemble probabilities, and visualizes the final decision at the selected threshold

The developed 2-stage system was supported by a web-based interface using Streamlit framework (Snowflake Inc., USA) for clinical usability and accessibility. The application was optimized to run on Google Colab environment and supports single or batch image processing modes. Users can upload panoramic radiographs via drag-and-drop method; the system automatically performs YOLO-based tooth detection and ensemble CNN classification, presenting results in visual and numerical formats. The user interface and example results are shown in Fig. 2. The interface includes real-time progress indicators, detailed JSON format reporting, and publication-quality visualization features. All processes can be transparently monitored through progress bars and instant feedback mechanisms. Intended end-users are general dentists and endodontists; the interface requires no specialized informatics expertise beyond routine radiograph interpretation.

### Statistical analysis

Statistical analyses were performed using Python (v3.10) and SPSS (v27.0, IBM Corp., Armonk, NY, USA) software.

Python analysis: Confusion matrix, accuracy, sensitivity, specificity, precision, and F1-score were calculated using Scikit-learn library. Performance of seven different model configurations was systematically evaluated on 188 panoramic test images. Data manipulation and Excel format reporting were performed with Pandas, confusion matrix visualizations with Matplotlib and Seaborn. Missing data due to YOLO detection failure were systematically processed; CNN classification analysis was not performed in these cases and calculations were made only on samples with valid detections.

SPSS analysis: AUC values, standard errors, and 95% confidence intervals were calculated for each model using ROC curve analysis module. In the analysis, actual labels (C-canal presence: 0 = absent, 1 = present) were used as state variable, and model confidence scores (continuous variable in 0–1 range) as test variable. Separate ROC analyses were performed for #37 and #47 teeth.

DeLong test: Statistical significance of AUC differences between models was evaluated using DeLong test with scipy and statsmodels libraries in Python. DeLong test is a non-parametric method used to compare two dependent ROC curves, allowing objective comparison of different models’ discriminative power on the same dataset. Comparisons were made for all pairwise combinations among seven models (total 21 pairwise comparisons: 7C2 = 21); z-score and p-value were calculated for each comparison. All ROC curve analyses and DeLong comparisons were performed on a per-tooth basis, where each mandibular second molar (#37 and #47) was treated as an independent unit of analysis.The 95% confidence intervals (CI) for individual AUC values and for pairwise AUC differences were calculated using the DeLong nonparametric method, which accounts for covariance between correlated ROC curves derived from the same set of observations.

Multiple comparison correction: Bonferroni correction was applied to control Type I error rate. Significance level was set at α = 0.05; corrected alpha value for 21 comparisons was calculated as α = 0.05/21 = 0.0024. *P* < 0.05 was considered statistically significant, *P* < 0.0024 as significant after Bonferroni correction.

### Handling of indeterminate/missing results

Cases where the YOLO model could not detect teeth or confidence score fell below threshold were recorded as “detection failure” and CNN classification stage was not proceeded. These cases were reported within total failure rate; however, they were excluded from denominator in calculating CNN model performance metrics. Similarly, cases where CNN model produced uncertain predictions (confidence score ≈ 0.5) were recorded separately; however, binary classification threshold (0.5) was applied to classify these cases as positive or negative. All missing and uncertain results will be transparently reported in the results section.

## Results

### Inter-rater reliability for reference standard

During the bounding box labeling process for mandibular second molars (#37 and #47) across 1.252 images, disagreement between 2 experienced endodontists occurred in only 7 cases (0.56%). These cases were resolved through consensus with a third expert. Complete agreement was achieved in all remaining cases, yielding excellent inter-rater reliability (κ = 0.99). For CBCT-based C-shaped canal labeling, two experienced endodontists independently evaluated all cases. Disagreements were resolved by consensus with a third expert. Inter-observer agreement was almost perfect (Cohen’s κ = 0.997).

### Stage 1: automatic tooth detection performance (YOLOv8)

The YOLOv8 model demonstrated exceptional performance in automatically localizing mandibular second molars. Training convergence curves (shown in Supplementary Fig. S1/S2) revealed consistent improvement across all metrics, with precision-recall curves indicating high selectivity and sensitivity simultaneously. Loss functions (box, classification, and distribution focal loss) decreased steadily without overfitting, and mAP@50 and mAP@50–95 metrics reached approximately 90%.

On the independent test set of 188 panoramic radiographs, the model achieved 100% accuracy for tooth #37 (187 true positives, 1 false positive, 0 false negatives) and 97.9% accuracy for tooth #47 (184 true positives, 5 false positives, 4 false negatives), yielding overall metrics of 98.40% precision, 98.93% recall, 98.66% F1-score, and 98.9% accuracy.

Notably, model performance remained completely stable across IoU thresholds from 0.5 to 0.85, with mAP consistently maintained at 0.9091 (Supplementary Fig. S3). Even under stringent thresholds, mAP reached 0.8182 at IoU 0.9 and 0.7273 at IoU 0.95.

### Stage 2: C-shaped canal classification performance

The tertiary ensemble CNN demonstrated strong performance on the isolated cropped tooth test set (*n* = 340). Training curves (shown in Supplementary Fig. S4) showed rapid convergence, with accuracy, F1-score, and sensitivity surpassing 95% after epoch 25 and remaining stable throughout 100 epochs. Final performance metrics on this standardized test set were: accuracy 99.4%, sensitivity 100%, specificity 99.0%, precision 98.6%, and F1-score 99.3%.

#### Integrated two-stage pipeline analysis

The end-to-end pipeline (YOLOv8 tooth detection + CNN classification) achieved high accuracy and reliability across both stages.

### Detection stage results

YOLOv8 successfully detected 369 of 373 target teeth, with only 4 detection failures (98.93% success rate). Six redundant bounding boxes for the same teeth were excluded from analysis. The small number of missed detections and false positives provided a reliable foundation for the classification stage. Four cases with failed or erroneous ROI extractions were manually corrected before CNN classification.

#### Classification stage results

CNN-based classification was performed on the 369 valid tooth crops obtained by YOLO. Table 1 presents comprehensive comparative performance of all seven model configurations.

**Table 1:** Comparison of C channel detection performances of CNN-based models and ensemble configurations in panoramic radiographs

Model	Accuracy	Precision	Recall	F1-Score	Specificity	TP	TN	FP	FN
ConvNeXt	0.9702	0.9671	0.9608	0.9639	0.9769	147	211	5	6
ConvNeXt-EfficientNet	0.9621	0.9542	0.9542	0.9542	0.9676	146	209	7	7
DenseNet-ConvNeXt-EfficientNet	0.9593	0.9481	0.9542	0.9511	0.9630	146	208	8	7
EfficientNet	0.9539	0.9474	0.9412	0.9443	0.9630	144	208	8	9
DenseNet-ConvNeXt	0.9539	0.9474	0.9412	0.9443	0.9630	144	208	8	9
DenseNet-EfficientNet	0.9539	0.9474	0.9412	0.9443	0.9630	144	208	8	9
DenseNet	0.9431	0.9400	0.9216	0.9307	0.9583	141	207	9	12

ConvNeXt achieved the highest overall performance with 97.02% accuracy, 96.08% sensitivity, and 96.39% F1-score. False negatives were limited to only 6 cases, with 5 false positives. This single architecture demonstrated that ensemble approaches were not necessary to achieve excellent performance.

EfficientNet-ConvNeXt (50%–50%) binary ensemble ranked second, with accuracy and sensitivity very close to ConvNeXt alone (96.21% accuracy, 95.42% sensitivity). This configuration recorded 7 false negatives and 7 false positives.

Tertiary ensemble (DenseNet-169-ConvNeXt-EfficientNet, 35%−35%−30%) demonstrated balanced performance with 95.93% accuracy and 95.42% sensitivity. DenseNet-169, EfficientNet-B6, and their binary ensemble combinations also achieved high accuracy (94.3–95.4%), but sensitivity values remained in the 92–94% range, trailing the leading models. DenseNet-169 alone showed the lowest performance (92.2% recall, 12 false negatives). The ensemble weight ratios (DenseNet-169: 35%, ConvNeXt-Base: 35%, EfficientNet-B6: 30%) were determined empirically based on preliminary validation experiments to ensure balanced contributions from each model. Several alternative weighting configurations were explored during preliminary testing; however, these variations did not result in meaningful improvements in overall performance. Therefore, the selected weight distribution was used in the final ensemble model.

A brief error analysis was conducted to examine false positive and false negative predictions across the evaluated models. False negatives were mainly observed in teeth presenting subtle or incomplete radiographic features of C-shaped canal morphology, where root fusion patterns were less pronounced on panoramic projections. False positive predictions were occasionally associated with radiographic appearances such as overlapping trabecular structures or root superimposition that mimicked fused root morphology. No clear clustering of errors was observed between mandibular second molars #37 and #47, indicating comparable model performance across both tooth positions.

#### Tooth-specific analysis

Among 153 C-shaped canal positive samples, 78 were from tooth #37 and 75 from tooth #47. Tooth-specific analysis clearly demonstrated ConvNeXt’s leadership:

- **Tooth #37**: ConvNeXt 96.2% sensitivity (75/78), EffNet-ConvNeXt 96.2% (75/78), tertiary ensemble 94.9% (74/78).
- **Tooth #47**: ConvNeXt 96.0% sensitivity (72/75), tertiary ensemble 96.0% (72/75), EffNet-ConvNeXt 94.7% (71/75). DenseNet-169 showed the lowest result for this tooth at 90.7% (68/75).

**Fig. 3:**
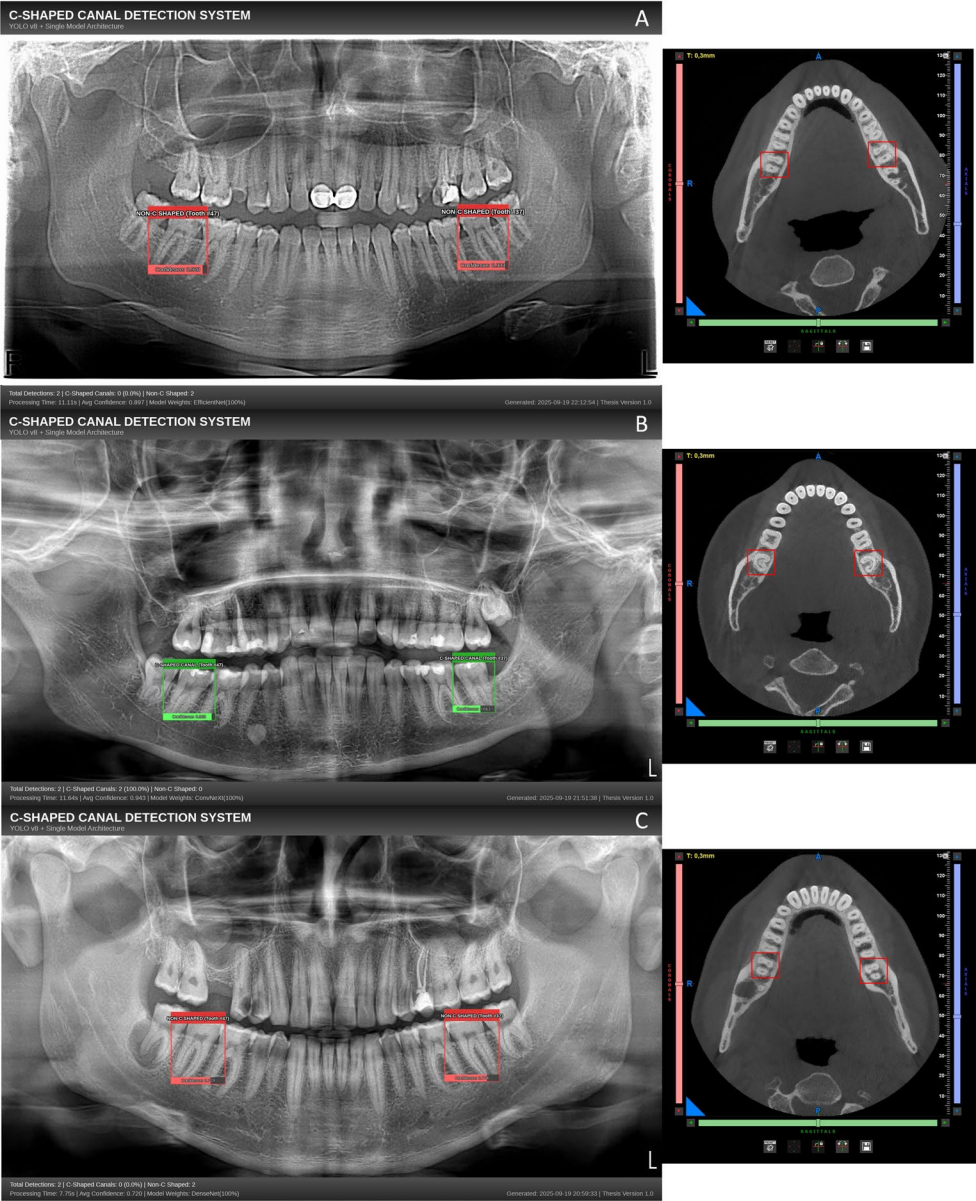
Model outputs on panoramic radiographs (left) and the corresponding CBCT horizontal slices used as the reference standard (right). (**A**) EfficientNet-B6, (**B**) ConvNeXt-Base, and (**C**) DenseNet-169 classifiers. On the panoramic images, green boxes indicate the presence of a C-shaped canal and red boxes indicate its absence; all boxes reflect YOLOv8-based tooth localization. On the CBCT panels, the red boxes delineate the mandibular second molars (#37 and #47) used for ground-truth adjudication

**Fig. 4:**
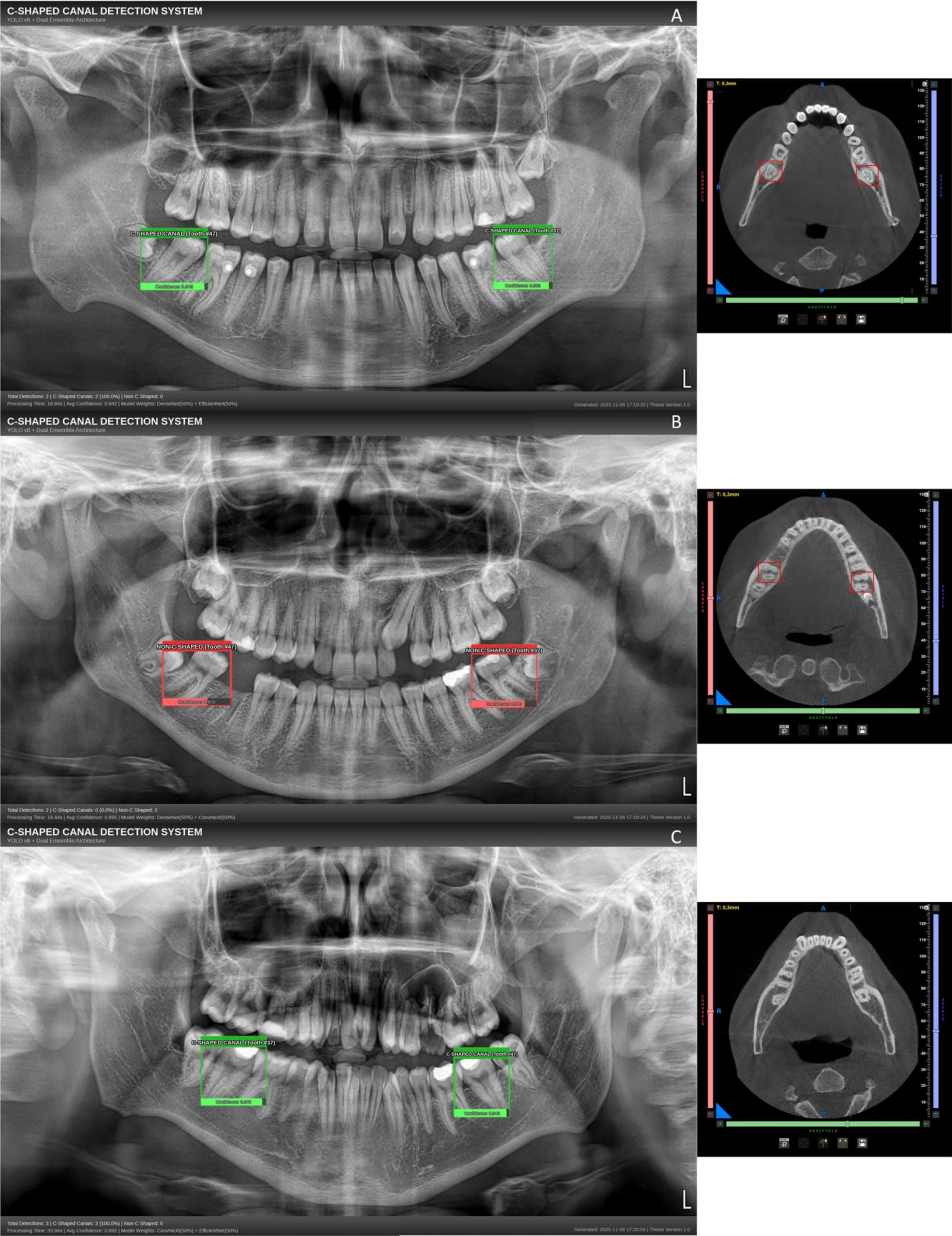
Dual ensemble classifier outputs on panoramic radiographs (left) with the corresponding CBCT horizontal slices used as the reference standard (right). (**A**) DenseNet-169 + EfficientNet-B6, (**B**) DenseNet-169 + ConvNeXt-Base, (**C**) ConvNeXt-Base + EfficientNet-B6. On the panoramic images, green boxes indicate the presence and red boxes the absence of a C-shaped canal; all boxes reflect YOLOv8-based localization of the target teeth. On the CBCT panels, the red boxes delineate the mandibular second molars (#37 and #47) used for ground-truth adjudication

Figures 3 and 4 illustrate representative detection examples from different pipeline models, demonstrating successful C-shaped canal identification and classification across diverse radiographic presentations.

#### ROC analysis and statistical comparisons

AUC values for seven model configurations were compared pairwise using DeLong test. Table 2 summarizes tooth-specific AUC values for all models.

**Table 2:** Area under the ROC curve (AUC) values for C-shaped canal detection performance of the models in tooth #37 and #47

Model	#37-AUC (95% CI)	#47-AUC (95% CI)
ConvNeXt	0.9735 (0.943–1.000	0.9881 (0.978–0.998)
DenseNet	0.9264 (0.885–0.967)	0.9164 (0.874–0.959)
EfficientNet	0.9447 (0.910–0.980)	0.9509 (0.921–0.981)
DenseNet-ConvNeXt	0.9535 (0.907–1.000)	0.9548 (0.925–0.985)
DenseNet-EfficientNet	0.9610 (0.928–0.994)	0.9644 (0.930–0.999)
ConvNeXt-EfficientNet	0.9730 (0.945–1.000)	0.9702 (0.947–0.993)
DenseNet-ConvNeXt-EfficientNet	0.9636 (0.932–0.995)	0.9622 (0.935–0.989)

##### For tooth #37

ConvNeXt demonstrated the highest AUC (0.9735), followed by Eff-Conv (0.9730), Ensemble_3 (0.9636), Dense-EffNet (0.9610), Dense-Conv (0.9535), EfficientNet (0.9447), and DenseNet-169 (0.9264).

##### For tooth #47

ConvNeXt again achieved the highest performance (0.9881), followed by Eff-Conv (0.9702), Dense-EffNet (0.9644), Ensemble_3 (0.9622), Dense-Conv (0.9548), EfficientNet (0.9509), and DenseNet-169 (0.9164).

To control Type I error risk from multiple comparisons, Bonferroni correction was applied (α = 0.05/21 = 0.0024). In preliminary analysis at α = 0.05, ConvNeXt was significantly superior to DenseNet-169 for tooth #37 (*p* = 0.036), and significant differences were observed in six model pairs for tooth #47. After Bonferroni correction, only ConvNeXt’s superiority over DenseNet-169 for tooth #47 remained statistically significant (AUC 0.988 vs. 0.916, *P* < 0.001). For tooth #37, inter-model differences did not reach statistical significance after correction.

These findings demonstrate that ConvNeXt provides sufficient performance as a single architecture without requiring ensemble approaches, and shows statistically proven superiority particularly for tooth #47 detection.

**Fig. 5:**
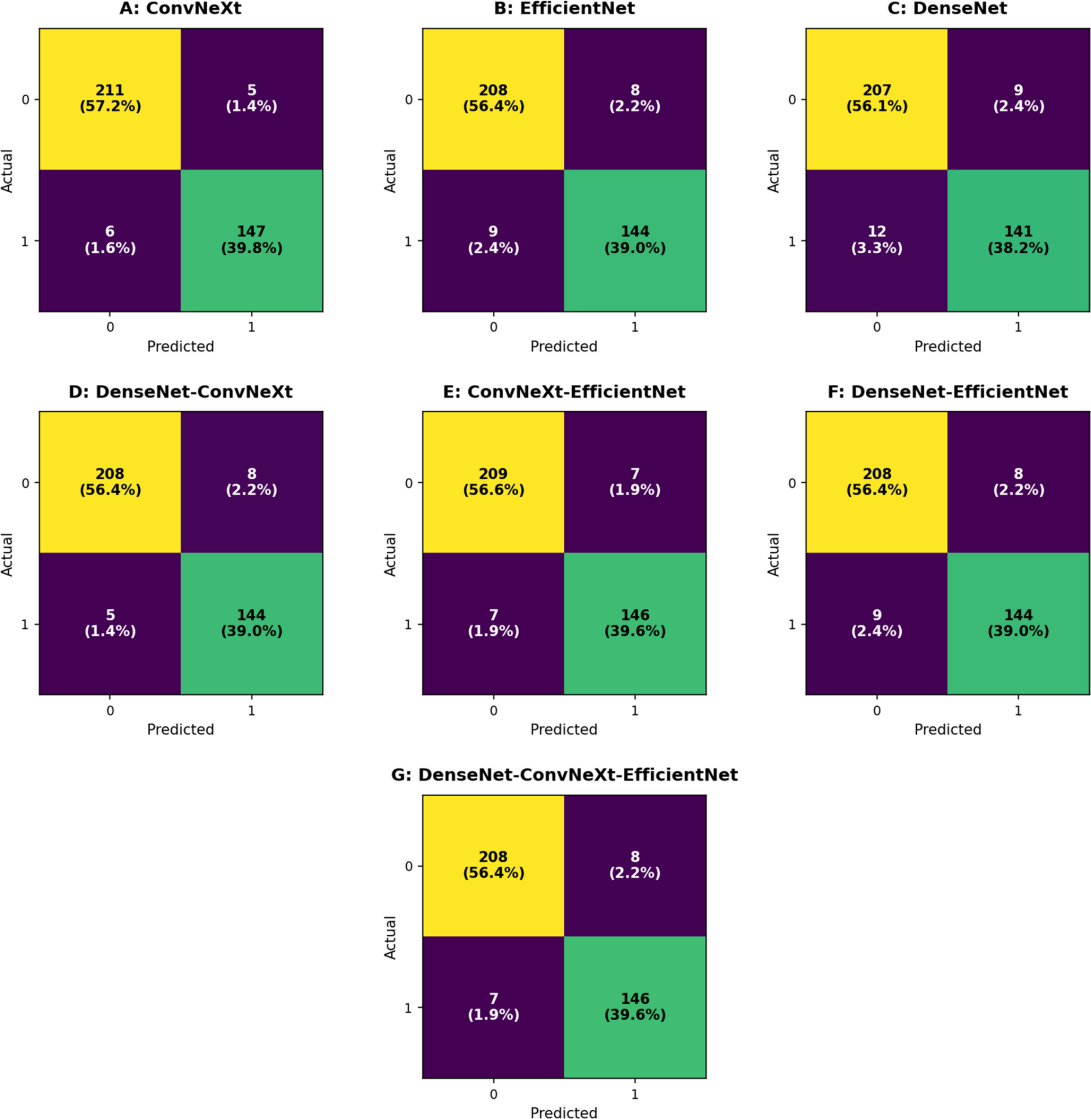
Confusion matrices for the CNN-based pipeline configurations. Shown are single-model classifiers—(**A**) ConvNeXt, (**B**) EfficientNet-B6, (**C**) DenseNet-169; two-model ensembles—(**D**) DenseNet-169–ConvNeXt (50%–50%), (**E**) EfficientNet–ConvNeXt (50%–50%), (**F**) DenseNet-169–EfficientNet (50%–50%); and the three-model ensemble—(**G**) DenseNet-169–EfficientNet–ConvNeXt. Color intensity reflects the distribution of correct (True Positive/True Negative) and incorrect (False Positive/False Negative) classifications

Figure 5 presents confusion matrices for single, binary, and tertiary CNN model combinations, visually comparing each model’s performance in distinguishing C-shaped canal positive and negative teeth.

## Discussion

C-shaped canal morphology represents one of the most challenging anatomical variations in endodontic treatment, featuring fused or interconnected canals that render conventional shaping methods inadequate [20]. This complex structure prevents rotary files and irrigation solutions from reaching the entire canal system, complicating thorough debridement and hermetic obturation [21]. Additionally, critically thin dentin regions create elevated strip perforation risk, while residual tissues and unfilled voids can perpetuate infection, leading to treatment failure20. Early detection of C-shaped canal variants is therefore critically important for planning appropriate treatment protocols and improving success rates.

When C-shaped morphology is identified pre-treatment, conservative root canal preparation can be adopted, instrument use minimized on invaginated surfaces to reduce perforation risk, and specialized techniques employed-including ultrasonically activated irrigation for cleaning and thermoplastic gutta-percha for obturation [21].

However, pre-treatment identification faces substantial obstacles due to inherent limitations of 2D imaging. Periapical and panoramic radiographs prove inadequate in reflecting 3D complex anatomical structures on a single plane. Cooke & Cox [9] reported that C-shaped canal detection on routine pre-treatment radiographs is impossible. Bucco-lingual root fusion, ribbon-shaped canal structures, and fine canal connections are frequently overlooked due to superimposition, dense trabecular bone patterns, and contrast limitations [22, 23]. Fused roots may appear as normal separate roots, and canal anatomy appearing normal at pulp chamber level may mask complex apical structures, creating a misleading impression of normaly [22].

CBCT has become the gold standard by eliminating these diagnostic uncertainties, enabling direct visualization of C-shaped cross-sectional morphology in axial sections and detailed evaluation according to Fan classification [24, 25]. However, high radiation dose, cost, and accessibility constraints make routine diagnostic CBCT inappropriate- it should be reserved for indication-based use only [24, 25].

This diagnostic dilemma − 2D imaging inadequacy versus 3D imaging impracticality for routine screening- creates an urgent need for innovative solutions. AI applications aimed at extracting maximum diagnostic information from existing panoramic images are rapidly gaining importance in endodontics. The YOLOv8 + CNN-based hybrid system developed in this study addresses this gap by automatically detecting mandibular second molars and classifying C-shaped canal presence without manual intervention. On 2D panoramic radiographs, the model achieved 98.9% detection accuracy, 97.02% classification accuracy, and 96.08% sensitivity, demonstrating very high diagnostic performance from routine imaging without additional radiation exposure or cost.

Current literature [26] demonstrates that AI-based clinical decision support tools significantly improve diagnostic performance. AI-supported clinicians achieved 0.8537 sensitivity compared to 0.7672 in unsupported groups (*P* < 0.05), revealing that AI systems not only increase accuracy but also contribute to diagnostic process standardization. However, AI should be positioned as a supportive tool, not a replacement for clinical judgment [27].

Our hybrid model can automatically flag high-risk cases on panoramic radiographs, alerting clinicians to possible morphological anomalies. Clinicians can then integrate the model’s recommendations with clinical findings and patient characteristics to make evidence-based CBCT indication decisions. This approach aligns with ALARA-ALADA (As Low As Reasonably Achievable – As Low As Diagnostically Acceptable) principles by ensuring advanced imaging is used only when necessary and patient-specifically [26]. By offering early diagnosis, low cost, accessibility, and radiation safety, the developed AI-based system provides a powerful alternative that has potential to reduce unnecessary imaging and improve endodontic treatment outcomes through physician-AI collaboration.

Compared with previous panoramic radiograph–based studies [16, 17], the present work provides three main contributions: (1) the implementation of a fully automated two-stage pipeline integrating YOLO-based tooth detection with CNN-based C-shaped canal classification, eliminating manual cropping; (2) a systematic comparison of three advanced CNN architectures and multiple ensemble strategies within the same dataset; and (3) comprehensive statistical evaluation using ROC analysis and DeLong testing to objectively compare model performance. These elements aim to provide a more reproducible and clinically interpretable framework for AI-assisted detection of C-shaped canal morphology on panoramic radiographs.

The YOLO + CNN hybrid architecture was selected to address limitations of classification-only approaches. While most studies require manual tooth region determination [28, 30], our automated pipeline eliminates this preprocessing step, reducing operator dependence and enabling simultaneous bilateral analysis. This design aligns with Yang et al.‘s [30] finding that automatic cropping + classification workflows achieved superior performance (AUC 0.945) compared to manual approaches in 805 patients. Our model’s performance is comparable to leading studies in the field. Jeon et al. [31] reported 95.1% accuracy and 92.7% sensitivity with CNN-based C-shaped canal detection on panoramic radiographs, while Zhang et al. [17] achieved 98.4% accuracy and 0.996 AUC using lightweight CNN architectures. In our study, ConvNeXt demonstrated 97.02% accuracy, 96.08% sensitivity, 97.69% specificity, and 96.39% F1-score, supporting rejection of the H₀₂ hypothesis. YOLOv8 detection achieved 98.9% accuracy (369/373 mandibular second molars correctly detected) with mAP@[0.5:0.95] of 0.8818, supporting rejection of the H₀₁ hypothesis. Tooth-specific analysis confirmed consistent performance: #37 sensitivity 96.15% (75/78) and #47 sensitivity 96.0% (72/75). The EfficientNet-ConvNeXt binary ensemble produced results nearly identical to ConvNeXt alone (96.21% accuracy, 95.42% sensitivity), while the tertiary ensemble showed slight performance decline (95.93%), suggesting that carefully selected single architectures may be preferred for this specific task.

One of the important factors affecting model performance is the CNN architecture used and training dataset size. In this context, Jaiswal and colleagues’ study [32] on 620 panoramic radiographs compared 26 different CNN architectures including VGG, ResNet, Inception, MobileNet, DenseNet-169, and EfficientNet for primary and permanent teeth classification. Study results revealed that EfficientNet-B0 and EfficientNet-B3 models showed highest performance with 98% accuracy, precision, recall, and F1-score values. In another study, Sherwood et al. [28] compared 3 different architectures while performing C-shaped canal classification in 135 limited field-of-view CBCT images and showed that the Xception-based U-Net model provided significantly higher Dice score and sensitivity compared to basic U-Net architecture. This finding indicates that advanced CNN architectures may be more successful in detecting complex morphologies. Additionally, it has been reported that very complex models may be prone to overfitting without sufficiently large and diverse datasets.

At this point, transfer learning effectiveness makes it possible to develop robust models even with relatively small datasets, especially in domain-specific applications such as medical image analysis. Literature [33] has shown that successful results can be obtained by using pre-trained CNN models in dental images where manual feature extraction is difficult.

In our study, C-shaped canal detection was performed using 3 different powerful CNN architectures. Models were trained with transfer learning approach, and training examples were enriched by applying various data augmentation techniques such as rotation, reflection, and scaling. Through this strategy, the model’s generalization capability was increased and overfitting risk was minimized. Thus, our pipeline model working with the existing dataset achieved success levels in other studies in literature, reaching 97.02% accuracy and 96.08% sensitivity for ConvNeXt. Tooth-based analysis results proved that it exhibited consistent performance in both tooth types by showing 96.15% sensitivity (75/78) for tooth #37 and 96.0% sensitivity (72/75) for tooth #47.

In literature [28–30] various methods such as only classification models, first segmentation then classification, or integrated approaches with object detection are mostly used for C-shaped canal detection. For example, in a CBCT-based study [27], a U-Net derivative network was first used to segment root canal structure, then a classification stage was used to determine whether C-shaped canal was present.

Recent studies have also reported conceptually similar two-stage pipelines in which automatic detection modules are followed by classification algorithms while using CBCT as the reference standard for labeling panoramic radiographs. These studies support the feasibility of hybrid detection–classification frameworks for identifying complex root canal morphologies in routine radiographic imaging. In line with this emerging methodological trend, the present study implemented a fully automated two-stage pipeline combining YOLO-based tooth detection with CNN-based classification. However, unlike previous studies, we additionally performed a systematic comparison of multiple CNN architectures and ensemble strategies within the same dataset and evaluated model performance using comprehensive ROC and DeLong statistical analyses. These methodological features provide a more detailed evaluation of model performance and contribute to improving the reproducibility of AI-assisted C-shaped canal detection on panoramic radiographs [34, 35].

In another study, Yang et al. [30] compared three different workflows (manual cropping, automatic cropping + classification, single-step detection + classification) on 805 patients, and highest AUC values reached 0.945 with automatic cropping + classification method. Yang et al.‘s [29] study investigated the effect of image type and cropping strategy on C-shaped canal classification in detail, and in their study on 1000 images, highest AUC value of 0.99 was reached with combination of periapical and panoramic images, showing that image patches containing only root region gave better results.

In our approach, a strategy similar to one of the most successful approaches reported by Yang et al.30 automatic cropping and classification-based method, was followed. Relevant tooth regions in panoramic radiographs were detected and classified in a single stage using YOLO algorithm, eliminating the need for manual cropping or segmentation steps. Thanks to YOLO’s real-time object detection feature, multiple mandibular second molars can be analyzed simultaneously on a panoramic radiograph and C-shaped canal presence can be evaluated.

Literature has reported that YOLO-based models accelerate clinical workflow in tasks such as tooth detection and numbering [36]. Similarly, our hybrid model has the advantage of being able to scan the entire panoramic film and automatically flag teeth carrying C-shaped canal anomalies. This presents a more practical clinical decision support tool compared to models that only perform classification.

There is no clear consensus in literature on whether single CNN models or ensemble approaches are superior for C-shaped canal detection. Our study fills this gap by revealing that ConvNeXt single model showed highest performance with 97.02% accuracy, while EfficientNet-ConvNeXt ensemble gave very close results with 96.21% accuracy.

Remarkably, the ensemble approach did not provide expected superiority; in fact, performance dropped to 95.93% in the tertiary ensemble. These findings demonstrate that in specific dental imaging tasks such as C-shaped canal detection, correctly chosen single architectures can be preferred over ensembles. Our model contributes to filling the literature gap on single model-ensemble performance comparison by achieving high accuracy rates with YOLO + CNN hybrid approach while offering real-time and holistic analysis.

**Fig. 6:**
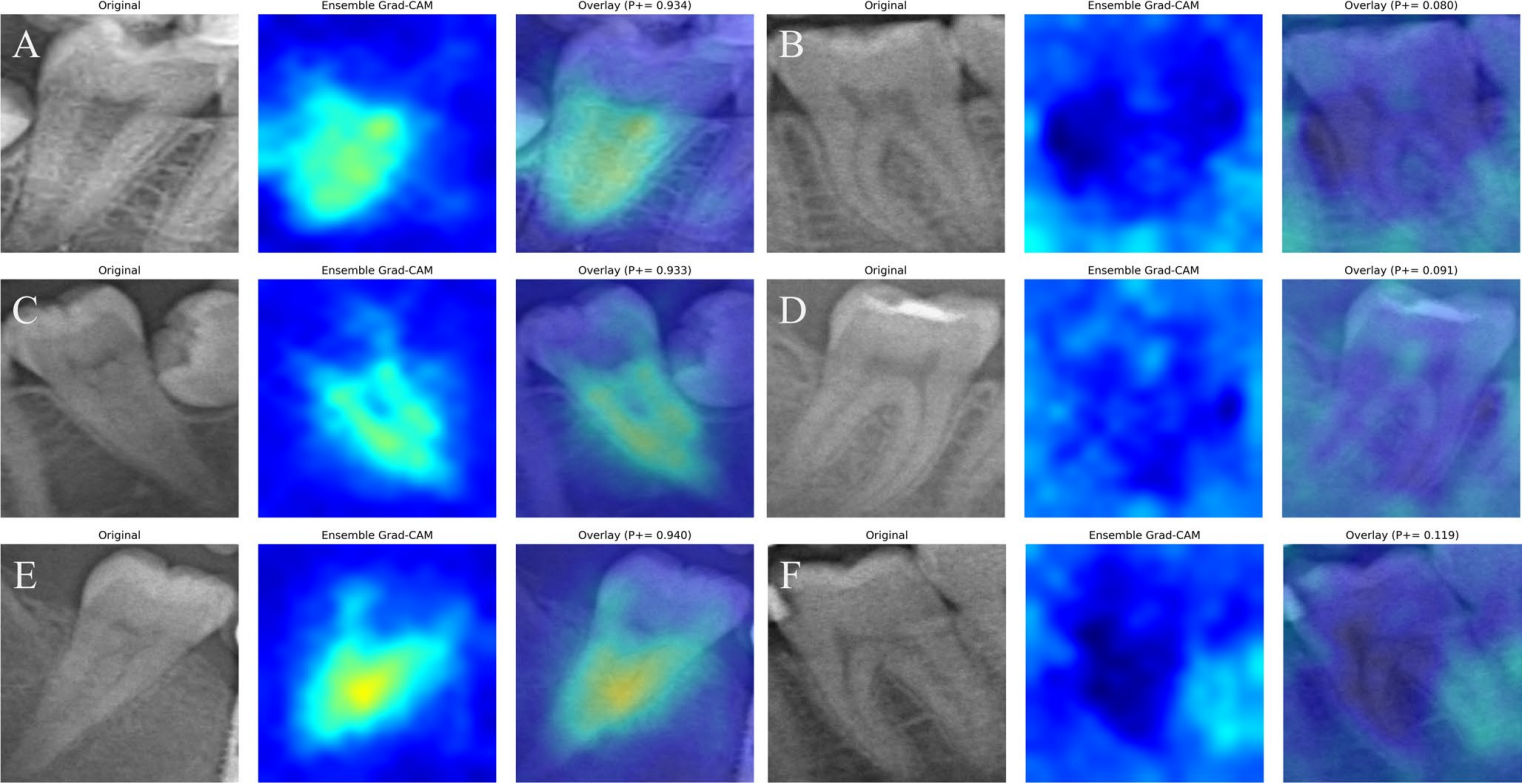
Grad-CAM visualizations for the ConvNeXt model in detecting C-shaped canal presence. (**A**, **C**, **E**) Positive cases showing strong activations over relevant anatomical regions; (**B**, **D**, **F**) negative cases exhibiting diffuse or low-intensity activation patterns

Integration of Gradient-weighted Class Activation Mapping (Grad-CAM) addresses a critical dimension of model transparency. Grad-CAM is a well-established post-hoc explainability technique for CNNs that has become increasingly essential in high-risk medical decision-making contexts [37]. Grad-CAM visualizations obtained in our study (Fig. 6) revealed anatomical regions the model prioritized during C-shaped canal detection. In positive cases (A, C, E), the model focused on characteristic conical root morphology with high confidence scores (P + = 0.934, 0.933, 0.940), generating activation heat maps concentrated on clinically relevant features. Conversely, negative cases (B, D, F) showed minimal activation patterns with low confidence scores (P + = 0.080, 0.091, 0.119), confirming correct identification of normal anatomy while avoiding false positives. In clinical use, Grad-CAM heatmaps may help clinicians verify whether the model’s prediction is based on anatomically plausible regions, such as the fused root morphology typically associated with C-shaped canals. Misclassified cases were also reviewed using Grad-CAM, which showed that some errors occurred when the model focused on adjacent anatomical structures or trabecular patterns rather than the root morphology itself. Such explainability approaches may improve transparency of AI systems and support clinician trust in AI-assisted diagnostic decision making.

Such explainability approaches are critical for clinical adoption, as the opaque nature of deep learning models can limit trust and usability [37]. By providing visual insights into decision processes, Grad-CAM enhances clinician understanding and confidence in AI-based diagnostic support. Literature demonstrates that explainable AI techniques like Grad-CAM significantly improve diagnostic reliability by making model reasoning transparent and highlighting pathology-relevant regions [37]. Our hybrid approach similarly provides both local and global interpretability, increasing clinical trust and applicability for detecting complex anatomical variations.

The sequential two-stage architecture adopted in the present study offers several methodological advantages compared with unified end-to-end models. By separating tooth localization and canal morphology classification, each stage of the pipeline can be optimized independently, improving flexibility during model development and evaluation. Detection-based cropping enables the CNN classifier to operate on standardized regions of interest (ROI), thereby reducing background interference from surrounding anatomical structures commonly present in panoramic radiographs. Modular AI pipelines have been widely adopted in medical imaging applications because they facilitate interpretability, stage-specific performance evaluation, and scalable clinical integration of diagnostic systems [18, 26, 38]. In dental imaging research, detection–classification frameworks have also been successfully applied for automated tooth localization and anatomical structure identification on panoramic radiographs [30, 36]. Nevertheless, sequential architectures may introduce the potential risk of error propagation between stages; however, the high detection accuracy achieved by the YOLO model in this study minimized this limitation.

This study has several limitations that present opportunities for future research. First, the dataset was collected from a single center, which, despite including images from four different panoramic devices, limits demographic diversity and generalizability. While device heterogeneity enhances model robustness to technical variations-consistent with literature showing that diverse data sources improve AI system performance [38] validation with multicenter datasets encompassing different geographical regions, ethnic populations, and institutional protocols remains essential for regulatory approval and clinical deployment.

Second, important methodological gaps exist in model optimization. Ensemble weight ratios (DenseNet-169 35%, ConvNeXt 35%, EfficientNet 30%) were determined empirically without systematic hyperparameter optimization techniques such as grid search, Bayesian optimization, or genetic algorithms. This absence suggests that ensemble performance may be suboptimal and could be improved through rigorous optimization frameworks. Similarly, Test Time Augmentation strategies were not comprehensively optimized, and explainability evaluation was limited to Grad-CAM without comparative analysis using LIME, SHAP, or attention visualization methods. These limitations constrain full understanding of model decision processes and may limit clinical trust.

Third, the very high inter-observer agreement (κ = 0.99) for tooth localization, while reflecting anatomically distinct boundaries, may indicate minimal representation of borderline cases with ambiguous anatomy or challenging positioning in the dataset. Additionally, natural image quality variations (contrast, resolution, positioning errors) and inherent C-shaped canal anatomical complexity can affect model performance in real-world clinical settings. Another limitation of the proposed pipeline is that a small number of YOLO detection failures required manual correction of bounding boxes before the classification stage. This occurred in only four cases (< 0.5% of the dataset). Although the correction was limited to region localization and did not affect the diagnostic labels, it indicates that the current system still requires minimal human intervention. Future improvements in object detection performance may enable a fully automated pipeline.

Another methodological consideration relates to the unit of analysis. In the present study, ROC analyses and DeLong comparisons were performed on a per-tooth basis, treating each mandibular second molar (#37 and #47) as an independent observation. However, bilateral teeth originating from the same patient may not be fully statistically independent. In our dataset, 181 patients presented with bilateral mandibular second molars, and 93.9% showed concordant C-shaped canal status, corresponding to substantial within-patient agreement (Cohen’s κ = 0.874). Although this high concordance suggests strong anatomical symmetry, the per-tooth analytical approach may still slightly underestimate the variance of AUC estimates and DeLong test statistics. Future studies applying clustered ROC analysis methods (e.g., Obuchowski approach) may further address this potential correlation structure.

Future research should prioritize multicenter validation studies to establish robust performance across diverse protocols and populations, integration with 3D imaging modalities such as CBCT to enhance diagnostic accuracy, and expansion of model capacity to detect other complex anatomical variations including MB1, MB2, and curved canal configurations. Advanced hyperparameter optimization, comprehensive TTA evaluation, and multi-method explainability analysis (Grad-CAM, LIME, SHAP) will further enhance clinical utility. Development of real-time deployment algorithms and creation of a comprehensive AI ecosystem for endodontic diagnosis represent long-term goals that will require collaborative multicenter efforts and regulatory pathway development.

## Conclusions

This study developed and validated a two-stage artificial intelligence system integrating YOLOv8-based automatic tooth detection with convolutional neural network architectures for the classification of C-shaped canal morphology on panoramic radiographs. The system achieved high accuracy in both tooth detection and C-shaped canal classification, with ConvNeXt demonstrating the most consistent performance among the evaluated architectures. The binary ensemble approach showed performance comparable to the single ConvNeXt model, while the tertiary ensemble did not provide additional benefit. From a clinical perspective, the proposed system could function as a screening tool during routine panoramic radiographic evaluation, helping clinicians identify teeth with a high probability of C-shaped canal morphology before treatment. Such information may support diagnostic decision-making and assist clinicians in identifying cases where additional CBCT imaging may be clinically justified. Although multicenter validation is required, this automated pipeline demonstrates potential as a clinical decision-support tool in endodontic treatment planning.

## Supplementary Information

Below is the link to the electronic supplementary material.

Supplementary Material 1 (DOCX 35.9 KB)

Supplementary Material 2 (DOCX 935 KB)

Supplementary Material 3 (RAR 15.0 MB)

Supplementary Material 4 (RAR 39.1 KB)

## Data Availability

The raw radiographic datasets analyzed in this study contain potentially identifiable patient information and cannot be made publicly available due to institutional and ethical restrictions. De-identified sample images, annotation files, model configuration files, and analysis scripts are provided as Supplementary Materials. The complete codebase, training logs, and documentation are included in the Supplementary File. Additional materials are available from the corresponding author on reasonable request and with appropriate institutional approval.

## Code Availability

All code relevant to model training and evaluation is included in the Supplementary File.
