# Supplementary material for: Automated detection of C-shaped canals in mandibular second molars from panoramic radiographs: comparing single and ensemble convolutional neural networks within a 2-stage pipeline
Source: Clin Oral Investig. 2026 Apr 23;30(5):190. doi: 10.1007/s00784-026-06868-x (PMC13102809; doi:10.1007/s00784-026-06868-x)
Supplement: Supplementary file 1 — Supplementary Material 1 (DOCX 35.9 KB) [file 784_2026_6868_MOESM1_ESM.docx]

**Research Article, Clinical Oral Investigations**

**Automated Detection of C-Shaped Canals in Mandibular Second Molars from Panoramic Radiographs: Comparing Single and Ensemble Convolutional Neural Networks within a 2-Stage Pipeline**

Yunus Emre Çakmak, Kürşat Er

Department of Endodontics, Faculty of Dentistry, Akdeniz University, Antalya, Türkiye

**Corresponding Author:**

Yunus Emre Çakmak, DDS

Department of Endodontics, Faculty of Dentistry, Akdeniz University, Dumlupınar Bulv., 07058 Campus, Antalya, Türkiye

**Email:** dt.emrecakmak@gmail.com

**S1. Panoramic Radiography Equipment and Acquisition Parameters**

Panoramic radiographs used in this study were acquired using four different digital panoramic radiography systems routinely operated at …………… University Faculty of Dentistry. These devices represent commonly used clinical equipment from major manufacturers, providing a diverse technical baseline that enhances the generalizability of the developed artificial intelligence models. Importantly, the specific device used for each individual radiograph was not systematically documented in the digital archive system, precluding device-based stratification during analysis. This absence of device-specific metadata naturally contributed to the blinding principle, as neither the reference standard evaluators nor the model developers were aware of acquisition device identity, thereby preventing potential device-related bias in labeling or model optimization.

The four panoramic systems employed different acquisition parameters within clinically acceptable ranges, as specified by their respective manufacturers. These variations in kilovoltage peak (kVp), tube current (mA), and exposure time reflect the different engineering approaches to image optimization across manufacturers, yet all devices were operated according to their standard clinical protocols to ensure diagnostic-quality images. The technical specifications for each device are summarized in Table S1.

Table S1. Technical specifications of panoramic radiography devices

| Device model | kV (kilovoltage) | mA (milliamperes) | Exposure time (s) |
| --- | --- | --- | --- |
| Planmeca ProMax Dimax 4 (Planmeca, Finland) | 64 | 7 | 16 |
| KaVo OP 3D Pro (KaVo, Germany) | 66 | 9 | 16 |
| Carestream CS 8100SC (Carestream Health, USA) | 68 | 8 | 10.8 |
| Morita VeraView IC5 (J. Morita MFG. Corp., Japan) | 70 | 7.5 | 10 |

All devices produce 8-bit grayscale JPEG images with consistent pixel dimensions of 1914×1024 pixels. The variation in acquisition parameters across devices introduces a realistic heterogeneity in image characteristics that better reflects real-world clinical scenarios compared to single-device studies. This technical diversity is particularly valuable for assessing model robustness, as the artificial intelligence system must learn generalizable anatomical features rather than device-specific image signatures.

**S2. Cone-Beam Computed Tomography Reference Standard and Validation Protocol**

In this study, the presence or absence of C-shaped canal anatomy was determined exclusively on CBCT and served as the reference standard for all included teeth. Eligibility required (i) a diagnostically adequate CBCT volume obtained for legitimate clinical indications (e.g., complex endodontic diagnosis, treatment planning, or pre-surgical assessment) and (ii) a corresponding panoramic radiograph for the same patient acquired within a defined interval (±12 months). Cases without an appropriate CBCT for the index tooth were not eligible.

All CBCT examinations were performed by a certified radiology technician using a Veraview X800 system (J. Morita MFG. Corp., Kyoto, Japan) under standardized institutional protocols and ALARA principles. Typical parameters were 99 kVp, 4.8 mA, and 35.8 s exposure per acquisition. Two field-of-view (FOV) settings were used according to clinical need: a large FOV (15 × 15 × 14.1 cm) for broader assessments and a focused FOV (4 × 4 × 4 cm) when a limited region/tooth-specific evaluation was required.

For C-shaped canal adjudication, only mandibular second molars were evaluated. A predefined reading protocol was applied: two experienced endodontists (each with ≥5 years of clinical experience), blinded to the panoramic images and all model outputs, independently reviewed CBCT axial slices at 0.5-mm intervals from the cemento-enamel junction to the apex; coronal and sagittal reconstructions were consulted when necessary. C-shaped canal morphology was confirmed when a continuous fin-like or C-shaped lumen pattern was identified at least at one level, in accordance with the diagnostic criteria of Fan et al. Because the CBCT appearances were unequivocal under this binary (present/absent) reference standard, no discrepancies occurred, no third-reader adjudication was required, and inter-rater agreement was perfect (Cohen’s κ = 1.00; 100% agreement).

CBCT determinations (present/absent) were used to define the binary ground-truth labels for the panoramic radiograph dataset. CBCT images were not used as inputs for model training or testing. Panoramic readers and model developers were masked to CBCT findings during model development to prevent information leakage.

By restricting inclusion to teeth with a clinically acquired CBCT and applying a standardized, blinded reading protocol, we ensured a uniform, high-confidence reference standard across the entire cohort. This design aligns with STARD-AI recommendations for transparent reference standard reporting and avoids biases that can arise when labels are assigned from 2D images alone.

It is important to emphasize that CBCT-based reference standard determination was performed for ALL teeth included in the dataset—both training (1,916 teeth) and test sets (340 teeth). Every single tooth analyzed by the AI models had a corresponding CBCT evaluation that served as the ground truth label, ensuring uniform reference standard quality throughout the study.

**S3. Training Dataset Composition and Manual Correction Procedures**

The convolutional neural network (CNN) models for C-shaped canal classification were trained on cropped tooth images automatically extracted from panoramic radiographs. Following the initial YOLOv8-based tooth detection stage, regions of interest (ROI) corresponding to mandibular second molars were cropped from the full panoramic images and saved as individual files. This automated extraction process generated a large dataset of tooth-level images suitable for training classification models.

However, as with any automated detection system, the YOLOv8 model occasionally produced imperfect bounding box predictions due to challenging cases involving unusual anatomical presentations, overlapping structures, or suboptimal image quality. To ensure the highest quality training data for the subsequent classification stage, all automatically generated crops were manually reviewed by experienced endodontists. Cases where the bounding box failed to adequately encompass the target tooth, included excessive extraneous anatomy, or was incorrectly positioned were identified and manually corrected. This quality control step involved adjusting bounding box coordinates and re-cropping the images to ensure that each training sample contained a properly centered and adequately framed mandibular second molar.

The final training dataset, after manual correction and quality assurance, consisted of 1,916 cropped tooth images. These images were distributed across two classes: 799 images positive for C-shaped canal morphology and 1,117 images without C-shaped canals. This class imbalance (approximately 42% positive vs. 58% negative) reflects a realistic prevalence estimate for C-shaped canals in mandibular second molars within a mixed-ethnicity population, as reported in epidemiological literature. The stratified cross-validation approach employed during model training ensured that each fold maintained a similar class distribution, preventing the models from developing biases toward the majority class. The final training dataset consisted of 1,916 cropped tooth images derived from 1064 panoramic radiographs.

For testing purposes, a completely separate test set of 340 cropped tooth images was prepared using the same quality control procedures. This test set comprised 142 C-shaped canal positive cases and 198 negative cases, maintaining a similar class distribution to the training data. Critically, these test images were derived from the 188 panoramic radiographs that had been withheld from the training and validation cohorts, ensuring a rigorous evaluation of model generalization to entirely unseen cases.

**S4. YOLOv8 Architecture and Training Configuration Details**

The object detection component of the two-stage pipeline was implemented using the YOLOv8 architecture, specifically the medium variant (YOLOv8m), which offers a favorable balance between detection accuracy and computational efficiency. YOLOv8 represents the eighth major iteration of the You Only Look Once (YOLO) family of real-time object detectors, incorporating architectural refinements such as an anchor-free detection head, improved feature pyramid network, and enhanced loss functions compared to earlier versions. The medium variant was selected over lighter (nano, small) or heavier (large, extra-large) versions based on preliminary experiments suggesting optimal performance for the dental radiograph detection task at this scale.

Transfer learning was employed to leverage knowledge from a large-scale natural image dataset. The model was initialized with weights pre-trained on the Microsoft COCO (Common Objects in Context) dataset, which contains over 200,000 images spanning 80 common object categories. Although the visual domain of natural images differs substantially from dental radiographs, the low- and mid-level features learned during COCO pre-training (edges, textures, shapes) provide a useful initialization that accelerates convergence and improves final performance compared to training from random initialization. The entire network was then fine-tuned on the dental radiograph dataset, allowing all layers to adapt to the specific characteristics of panoramic images and tooth morphology.

All input images underwent standardized preprocessing before entering the network. The letterbox resizing technique was applied to transform the original 1914×1024 pixel panoramic radiographs into 640×640 pixel square inputs, which is the standard input resolution for YOLOv8. This resizing method preserves the original aspect ratio by padding the shorter dimension with black pixels, preventing distortion of anatomical structures that could occur with direct stretching or squashing. We used a 640×640 input—the YOLOv8 default—as a practical trade-off between detail and efficiency.

Training was conducted over a maximum of 100 epochs, with convergence typically achieved earlier due to the early stopping mechanism. Early stopping was implemented with a patience parameter of 20 epochs, meaning that training would terminate if no improvement in validation set mean average precision was observed for 20 consecutive epochs. This approach prevents overfitting to the training data by halting optimization before the model begins to memorize training-specific patterns at the expense of generalization.

The AdamW optimizer was selected due to its adaptive learning rate properties and effective weight decay regularization. The initial learning rate was set to 0.001, with gradual decay throughout training according to a cosine annealing schedule. This schedule smoothly reduces the learning rate from the initial value to near zero following a cosine curve, promoting stable convergence in later training stages. Weight decay was set to 5×10⁻⁴ to provide L2 regularization, and the momentum coefficient was maintained at 0.937 following default YOLOv8 recommendations.

The loss function comprised three weighted components addressing different aspects of object detection performance. The bounding box localization loss, which measures the accuracy of predicted box coordinates, was assigned a gain of 7.5, reflecting its critical importance in precisely locating teeth within the image. The classification loss, which measures the correctness of the predicted tooth class (tooth #37 vs. #47), was assigned a lower gain of 0.5 since distinguishing between the two tooth classes is relatively straightforward given their distinct spatial positions. Finally, the distribution focal loss (DFL), which encourages the model to produce sharp and confident bounding box predictions, was assigned a gain of 1.5. These relative weights were determined through preliminary experimentation to achieve optimal detection performance.

To further accelerate training and manage GPU memory efficiently, automatic mixed precision (AMP) training was enabled. This technique performs forward and backward passes using 16-bit floating-point precision where possible, falling back to 32-bit precision only where necessary to maintain numerical stability. The use of mixed precision approximately doubles training throughput on modern GPUs with tensor core hardware while maintaining equivalent model accuracy to full 32-bit training.

Data augmentation during YOLOv8 training focused on techniques appropriate for medical imaging that preserve anatomical plausibility. Mosaic augmentation, which combines four training images into a single composite image, was applied throughout most of the training to expose the model to diverse spatial configurations and improve robustness to scale and position variations. However, mosaic augmentation was disabled during the final 10 epochs to allow the model to fine-tune on individual, non-composite images and adapt to the true data distribution. Horizontal and vertical flipping were each applied with 50% probability, reflecting the bilateral symmetry of the mandible and the arbitrary orientation variability in patient positioning. Importantly, more aggressive augmentations such as mixup (blending multiple images) and copy-paste (inserting object instances from other images) were deliberately excluded, as these techniques can create anatomically implausible configurations that may confuse the model when applied to medical images.

At inference time, the trained model outputs multiple candidate detections per image, each with an associated confidence score. To filter false positives and retain only high-confidence detections, a confidence threshold of 0.25 was applied—detections with confidence below this value were discarded. Additionally, non-maximum suppression (NMS) was performed with an intersection-over-union (IoU) threshold of 0.5 to eliminate redundant overlapping detections of the same tooth, retaining only the highest-confidence detection per tooth. These threshold values were selected based on validation set performance to balance sensitivity (detecting all teeth) with precision (avoiding false detections).

**S5. Convolutional Neural Network Architecture Specifications**

Three distinct deep convolutional neural network architectures were employed for C-shaped canal classification, each offering unique inductive biases and feature learning capabilities. The selection of these specific models was motivated by their proven track records in medical image analysis tasks and their complementary strengths, which were hypothesized to produce robust ensemble performance when combined.

DenseNet-169 implements dense connectivity, wherein each layer receives direct connections from all preceding layers within a dense block. This architectural pattern promotes feature reuse, strengthens gradient flow during backpropagation, and encourages the network to learn diverse representations at different depths. The model processes input images at 224×224 pixel resolution and is initialized with ImageNet1K_V1 pre-trained weights, which encode general visual features learned from over one million natural images. All dense blocks were kept trainable during fine-tuning, allowing the network to adapt both low-level feature detectors and high-level semantic representations to the dental imaging domain. The classifier head consists of a sequence of fully connected layers that progressively reduce dimensionality: the 1664-dimensional global feature vector extracted from the final dense block is passed through layers with 768, 384, and finally 2 output neurons (corresponding to non-C-shaped and C-shaped canal classes). Dropout regularization with rate 0.5 is applied between fully connected layers to prevent overfitting, and batch normalization layers stabilize training dynamics. To manage GPU memory consumption given the model's depth, gradient checkpointing was enabled, trading modest computational overhead for substantial memory savings by recomputing intermediate activations during backpropagation rather than storing them.

ConvNeXt-Base represents a modernized purely convolutional architecture that incorporates design principles from vision transformers while maintaining the efficiency and scalability advantages of convolutional networks. Images are processed at 384×384 pixel resolution to preserve finer spatial details. The model employs GELU (Gaussian Error Linear Unit) activation functions throughout, which have been shown to provide smoother gradient landscapes compared to ReLU, and uses layer normalization rather than batch normalization for improved training stability. A key innovation is the integration of a channel attention module in the feature extraction pathway. This module computes attention weights across feature channels using adaptive average pooling followed by 1×1 convolutions and sigmoid activation, effectively allowing the network to dynamically emphasize the most informative feature channels for each input while suppressing less relevant ones. The classifier reduces the 1024-dimensional feature representation through intermediate layers of 768 and 384 dimensions before the final binary decision, with dropout regularization applied throughout.

EfficientNet-B6 employs compound scaling, simultaneously scaling network depth, width, and input resolution according to a principled coefficient that optimizes the trade-off between accuracy and computational cost. This model processes the highest resolution inputs at 528×528 pixels, enabling the finest-grained spatial feature extraction among the three architectures. Pre-trained initialization used the tf_efficientnet_b6_ns variant when available, which incorporates noisy student training—a semi-supervised learning method that further enhances representational quality. In cases where this specific pre-trained checkpoint was unavailable, the standard efficientnet_b6 architecture was used with random weight initialization, accepting slightly reduced initial performance in exchange for reliable model instantiation. A spatial attention mechanism is integrated into the architecture, which computes attention maps over the spatial dimensions of feature maps by combining average and maximum pooling operations, processing them through a 7×7 convolution, and applying sigmoid activation. This allows the model to focus on the most diagnostically relevant spatial regions (e.g., the root fusion area) while de-emphasizing irrelevant background regions. The classifier employs SiLU (Sigmoid Linear Unit, also known as Swish) activation, which has been shown to outperform ReLU in deep networks, and reduces the 2304-dimensional feature vector through intermediate layers of 1024 and 512 dimensions, with batch normalization applied for regularization.

All three models were trained using identical protocols to ensure fair comparison. Ten-fold stratified cross-validation was employed, wherein the training data was divided into ten approximately equal subsets (folds), each maintaining the original class distribution. In each of ten training iterations, nine folds were used for training and one fold for validation, rotating which fold served as validation across iterations. This approach produces ten independently trained models per architecture, allowing for robust performance estimation and quantification of model variability. Each fold was trained for a maximum of 100 epochs with early stopping implemented using a patience parameter of 25 epochs—training terminated if validation loss failed to improve for 25 consecutive epochs, preventing overfitting while allowing sufficient opportunity for convergence.

Mixed precision training (FP16) was enabled across all models to accelerate computation and reduce memory footprint on the NVIDIA A100 GPU. Batch sizes were independently optimized for each architecture based on GPU memory capacity and training efficiency: DenseNet-169 used batches of 48 images, ConvNeXt-Base used 32 images, and EfficientNet-B6 used 28 images. These batch sizes represent the maximum values that could be accommodated in GPU memory while maintaining stable gradient estimates; larger batches are generally preferable for optimization stability, but are constrained by hardware limitations. Dynamic batch size adjustment was implemented such that if a CUDA out-of-memory error occurred during training, the batch size would be automatically reduced and training resumed, ensuring robustness to memory availability variations.

The AdamW optimizer was used universally, with learning rates tuned per architecture based on preliminary experiments: DenseNet-169 and EfficientNet-B6 used 3×10⁻⁴, while ConvNeXt-Base used a slightly lower rate of 2×10⁻⁴ to account for its different normalization scheme. The CosineAnnealingWarmRestarts learning rate scheduler was applied with a restart period (T_0) of 10 epochs, causing the learning rate to periodically cycle from high to low values following a cosine curve. This cycling allows the model to escape local minima and potentially discover better solutions during later training phases. The minimum learning rate was set to 1/50 of the initial value to ensure continued small updates even at the end of long training runs. Gradient clipping with maximum norm 1.0 was applied to prevent exploding gradients, a common instability issue in deep networks, by rescaling gradients whose norm exceeds the threshold.

Beyond architectural and optimization choices, a sophisticated data augmentation pipeline was critical to model generalization. A comprehensive set of transformations was applied during training to artificially expand the effective dataset size and expose models to realistic variations they might encounter at test time. These augmentations, implemented using the Albumentations library, included geometric transformations (horizontal flip with 50% probability, vertical flip with 50% probability, random 90-degree rotation with 50% probability, and combined shift-scale-rotation with shift limit 0.15, scale limit 0.3, rotation limit 45 degrees, and application probability 80%), elastic deformations (elastic transform, grid distortion, and optical distortion, each with 50% probability to simulate subtle anatomical variations), pixel-level adjustments (brightness-contrast adjustment with 40% probability, contrast-limited adaptive histogram equalization with 80% probability, and HSV color space transformation with 80% probability despite grayscale images to model intensity variations), noise injection (Gaussian noise, Gaussian blur, and motion blur, each with 40% probability to simulate image acquisition artifacts), and coarse dropout (50% probability, randomly masking 16 rectangular regions of up to 32×32 pixels to encourage spatial invariance). Importantly, no augmentation was applied during validation or testing—images were simply center-cropped and normalized—to ensure performance metrics reflected true model capability on unmodified data.

Finally, to improve the reliability of probabilistic predictions, temperature scaling was applied as a post-processing calibration step. This technique involves learning a single scalar parameter (the temperature) that scales the logits (pre-softmax outputs) to better align predicted probabilities with empirical frequencies. Properly calibrated probabilities are crucial in clinical applications where the confidence of predictions informs decision-making. In ensemble configurations that combined two models (dual ensembles), a cross-attention mechanism was optionally implemented to facilitate feature fusion, allowing the models to dynamically weight their respective contributions based on the input characteristics. This attention mechanism was achieved through learned projection matrices that transform and combine the feature representations from both models before the final classification decision.

**S6. Grad-CAM Visualization for Model Interpretability**

To enhance the transparency and clinical interpretability of the classification models, Gradient-weighted Class Activation Mapping (Grad-CAM) was employed to visualize which regions of the input tooth images most strongly influenced model predictions. Deep neural networks are often criticized as "black boxes" that provide no insight into their decision-making process, raising concerns about trust and safety in clinical applications. Grad-CAM addresses this limitation by producing heatmaps that highlight the spatial regions most relevant to a particular class prediction, effectively showing where the model "looks" when making its decision.

The Grad-CAM technique operates by computing the gradient of the predicted class score with respect to the feature maps of the final convolutional layer. These gradients are then globally averaged to produce importance weights for each feature channel. The weighted combination of feature maps, followed by ReLU activation to retain only positive contributions, yields a coarse localization map that indicates which regions of the image contributed most strongly to the class prediction. This map is then upsampled to the original image resolution and overlaid as a heatmap, typically using a color scheme (e.g., red for high activation, blue for low activation) that makes important regions visually salient.

In the context of C-shaped canal detection, Grad-CAM visualizations served as a post-hoc validation mechanism to confirm that the models were focusing on clinically relevant anatomical features rather than spurious correlations or artifacts. For positive predictions (C-shaped canal present), the models were expected to activate on the root fusion zone, the longitudinal groove on the root surface, and the characteristic C-shaped lumen outline visible in the radiograph. For negative predictions (C-shaped canal absent), activation should be more diffuse or focused on distinct root separation zones. Manual review of Grad-CAM outputs by expert endodontists confirmed that the trained models exhibited attention patterns consistent with expert clinical reasoning, thereby bolstering confidence in the models' learned representations and providing a form of qualitative validation beyond quantitative accuracy metrics. These visualizations are exemplified in Figure 8 of the main manuscript, which illustrates the complete pipeline workflow including automatic tooth detection, classification, and interpretability visualization.

**S7. Pipeline System Architecture and Performance Optimization**

The complete C-shaped canal detection system integrates the YOLOv8 tooth detection module and the ensemble CNN classification module into a unified pipeline that processes full panoramic radiographs end-to-end without manual intervention. This two-stage architecture was specifically designed to enable fully automatic analysis while maintaining modularity—each stage can be independently evaluated, updated, or replaced without affecting the other. The pipeline was implemented in Python and organized into a series of functions that sequentially execute detection, cropping, classification, and result visualization.

At runtime, the system first loads the trained YOLOv8 model weights and initializes the GPU or CPU computational backend. When a panoramic radiograph is provided as input, it is preprocessed using the same letterbox resizing and normalization applied during training, then passed through the YOLOv8 network. The model outputs a list of detected bounding boxes with associated confidence scores and class labels (tooth #37 or #47). These detections are filtered using the pre-determined confidence threshold (0.25) and non-maximum suppression threshold (0.5) to eliminate low-confidence and duplicate detections. For each retained detection, the system extracts the corresponding image region by cropping according to the bounding box coordinates, producing an isolated tooth ROI.

Each cropped tooth image is then processed by the ensemble classification stage. The three CNN models (DenseNet-169, ConvNeXt-Base, EfficientNet-B6) are loaded into GPU memory, and the crop is resized to the appropriate input resolution for each model (224×224, 384×384, 528×528 pixels respectively). To further enhance prediction robustness, Test Time Augmentation (TTA) is applied: each model generates predictions for three different augmented versions of the input (original, horizontal flip, vertical flip), and the softmax outputs across these three predictions are averaged to produce a single probability distribution per model. The final ensemble prediction is computed as a weighted average of the three models' outputs. These weights (35% DenseNet, 35% ConvNeXt, 30% EfficientNet for the tertiary ensemble) were assigned based on observed model characteristics during cross-validation: EfficientNet-B6 exhibited higher inter-fold variance compared to DenseNet-169 and ConvNeXt-Base, suggesting lower stability, and was therefore assigned a slightly reduced weight to mitigate the potential impact of this variability on ensemble predictions.

Memory management and error handling are critical for reliable operation in real-world scenarios. The pipeline implements several strategies to ensure robustness. Batch size for both the detection and classification stages is dynamically adjusted based on available GPU memory—if a CUDA out-of-memory error is encountered, the system automatically reduces the batch size and retries the operation. If GPU resources are unavailable or exhausted, the pipeline can automatically fall back to CPU execution, trading speed for continued functionality. Model loading failures trigger fallback mechanisms, such as attempting to load alternative checkpoint files or switching to a default configuration. Visualization failures (e.g., missing fonts for text rendering) are handled gracefully by reverting to basic visualization without text annotations, ensuring that core functionality is preserved even when auxiliary features fail.

To facilitate batch processing of large datasets, the pipeline supports a batch mode wherein all images in a specified input directory are automatically processed in sequence. Results are saved both as annotated visualizations (with bounding boxes, labels, and confidence scores overlaid on the original panoramic image) and as structured data files. Each processed image produces a JSON file containing detailed detection and classification results: coordinates and confidence scores for each detected tooth, class probabilities from each model and the ensemble, final binary predictions, and processing timestamps. These JSON outputs enable downstream quantitative analysis and integration with electronic health record systems. Additionally, the batch mode generates a CSV summary file aggregating key statistics across all processed images, such as total number of detections, percentage of C-shaped canals detected, average confidence scores, and processing time per image. This summary facilitates rapid quality assessment and performance monitoring when applying the system to new datasets.

Processing speed is an important consideration for clinical deployment. On the NVIDIA A100 GPU used in this study, the average processing time per panoramic radiograph is approximately 20 seconds, encompassing tooth detection, cropping, ensemble classification, and result visualization. This corresponds to a throughput of roughly three images per minute, which is adequate for offline batch processing of accumulated cases but may be too slow for real-time clinical use during patient appointments. Future optimization efforts, such as model quantization, pruning, or deployment on specialized inference hardware, could reduce latency to enable interactive use cases. The pipeline's modular design facilitates such optimizations by allowing individual components to be replaced or upgraded without rewriting the entire system.

**S8. Web Application Development and Deployment**

To enhance accessibility and facilitate adoption by clinicians and researchers without extensive programming expertise, a web-based graphical user interface was developed using the Streamlit framework. Streamlit is an open-source Python library designed specifically for rapidly building interactive web applications for data science and machine learning projects, requiring minimal web development knowledge. The application provides an intuitive point-and-click interface for uploading panoramic radiographs, initiating analysis, and viewing results, thereby lowering the barrier to entry for users who may not be comfortable working directly with Python scripts or command-line tools.

The application architecture consists of a Python backend that invokes the complete two-stage pipeline and a Streamlit frontend that handles user interaction and result presentation. When launched, the application initializes by loading the pre-trained YOLOv8 model and the three CNN models into memory, which can take 30-60 seconds depending on hardware and model checkpoint sizes. Once initialization is complete, the user is presented with a file upload widget where they can drag and drop panoramic radiograph images in JPEG format. The application supports both single-image analysis and batch processing of multiple images simultaneously.

Upon upload, the backend processes each image through the pipeline as previously described. To keep the user informed of progress, real-time feedback mechanisms are implemented: a progress bar indicates the current processing stage (e.g., "Detecting teeth... 50% complete"), and live updates display intermediate results such as the number of teeth detected and preliminary classification probabilities. This transparency is particularly valuable for batch processing, where users may submit dozens of images and wish to monitor progress without manually checking each output file.

When processing is complete, results are presented through multiple visualization modalities. The primary output is an annotated version of the original panoramic radiograph with bounding boxes overlaid on detected teeth. Each bounding box is color-coded: green indicates a tooth classified as having a C-shaped canal, while red indicates absence of C-shaped morphology. Text labels adjacent to each box display the tooth number (#37 or #47), the predicted class, and the ensemble confidence score as a percentage. This visual summary allows clinicians to rapidly assess results at a glance. Additionally, the application provides detailed numeric output tables listing the exact probability values from each individual model and the ensemble, facilitating deeper analysis or quality auditing. For research purposes, a "Download Results" button allows users to export all results as a JSON file or a CSV table compatible with statistical software packages.

The application was deployed in headless mode, meaning it runs as a background server process without a graphical desktop environment, suitable for deployment on remote servers or cloud computing platforms. To maximize accessibility during development and testing, Cross-Origin Resource Sharing (CORS) and Cross-Site Request Forgery (XSRF) protections were disabled, allowing access from any client browser without authentication for research and development purposes only. These settings are appropriate solely for controlled research environments and must not be used in production deployments handling patient data. While appropriate for a controlled research environment, these settings would need to be tightened (e.g., implementing user authentication, enabling HTTPS encryption) before public deployment to ensure data security and patient privacy. The application server was started using the nohup command, which allows the process to continue running even after the terminal session is closed, and all console output is logged to a file for debugging and monitoring purposes.

The web application's convenience comes with some trade-offs. Because it runs on a shared server and models are loaded into GPU memory, only one user can effectively utilize the GPU at a time unless advanced scheduling mechanisms are implemented. Additionally, the application inherits the computational requirements of the underlying models, necessitating a server with a capable GPU to achieve acceptable processing speeds. Nevertheless, for research collaborations or institutional deployments where a dedicated server can be provisioned, the web interface significantly enhances usability compared to requiring users to install dependencies, configure environments, and execute command-line scripts.

**S9. Software Environment and Library Versions**

Reproducibility of computational research requires detailed documentation of the software environment in which experiments were conducted. All model development, training, and evaluation were performed using Python version 3.10, a stable release that balances modern language features with broad library compatibility. PyTorch version 2.0 served as the primary deep learning framework, providing efficient tensor operations, automatic differentiation, and GPU acceleration. PyTorch 2.0 introduced significant performance improvements through its torch.compile feature, which compiles model code into optimized execution graphs, although this feature was not explicitly enabled in the present work.

The YOLOv8 implementation was provided by the Ultralytics library, which offers a high-level API for training and deploying YOLO models. At the time of this study, the latest stable release of Ultralytics YOLOv8 was used (exact version numbers are subject to rapid updates; the version installed via pip at the time of experiments is recorded in the project's requirements.txt file). Data augmentation was implemented using the Albumentations library, a widely-used and actively-maintained package offering a comprehensive suite of image transformations optimized for performance and composability.

Scientific computing and data manipulation were handled by standard Python packages. NumPy provided low-level array operations and mathematical functions, while Pandas facilitated higher-level data manipulation, particularly for organizing performance metrics into tabular formats and exporting results to CSV and Excel files. Matplotlib and Seaborn were used for generating visualizations, including confusion matrices, ROC curves, and performance comparison plots. These libraries provide publication-quality plotting capabilities and integrate seamlessly with Jupyter notebooks, which were used for interactive exploration during model development.

Statistical analyses were conducted using a combination of Python and SPSS software. Within Python, the scikit-learn library provided implementations of fundamental machine learning utilities, including confusion matrix computation, precision-recall-F1 score calculation, and ROC curve generation. The SciPy library supplied statistical functions such as the DeLong test for comparing ROC curves, while the statsmodels library offered additional statistical modeling capabilities. SPSS Statistics version 28.0 (IBM Corporation, Armonk, NY, USA) was used for certain analyses, particularly ROC curve analysis with detailed output of standard errors and confidence intervals, and for generating formatted tables suitable for direct inclusion in manuscripts. The combination of Python's flexibility and SPSS's standardized reporting formats provided a robust analytical workflow.

The computational infrastructure consisted of Google Colab Pro+, a cloud-based Jupyter notebook environment offering access to high-performance GPUs. Specifically, the NVIDIA A100 GPU with 80 GB of VRAM was used for all training and inference tasks. Google Colab's integration with Google Drive allowed seamless data storage and retrieval; all datasets, model checkpoints, and results were stored in the researcher's personal Google Drive account, ensuring data security and accessibility. Importantly, no third-party cloud storage services were used, and data were not shared with external entities, maintaining compliance with institutional data protection policies. The Google Colab environment provides a pre-configured software stack including CUDA (the NVIDIA GPU computing platform) and cuDNN (the CUDA Deep Neural Network library), with versions compatible with the installed PyTorch version. At the time of these experiments, CUDA version 11.8 or 12.x was available depending on the specific Colab runtime instance, both of which are supported by PyTorch 2.0.

For annotation of training data, LabelImg version 1.8.6, a graphical image annotation tool, was used to manually draw bounding boxes around teeth in panoramic radiographs. LabelImg outputs annotations in XML format compatible with the PASCAL VOC standard, which were subsequently converted to YOLO format (normalized text files) using custom Python scripts. The institutional digital archive system, Metasoft Dentasist version 4.1.168 (Eskişehir, Turkey), served as the source of all panoramic radiograph images, providing a standardized interface for retrospective image retrieval.

Finally, the web-based user interface was built using Streamlit, an open-source framework for rapidly prototyping interactive machine learning applications. At the time of development, the latest stable release of Streamlit available via pip was used. Streamlit's reactive programming model automatically updates the user interface in response to user input, simplifying the development of dynamic web applications without requiring explicit management of state or HTML rendering.

This software ecosystem, while complex, represents a standard modern machine learning stack widely adopted across academic and industrial research. The use of popular, well-documented libraries enhances reproducibility, as other researchers can install identical environments using package management tools (pip, conda) and the requirements specification file accompanying the project code. Should discrepancies arise due to library updates or platform differences, the recorded version numbers allow precise reconstruction of the original environment.

**S10. Statistical Analysis Implementation Details**

The statistical analysis pipeline was designed to provide comprehensive evaluation of model performance across multiple metrics, with particular attention to rigorous hypothesis testing and correction for multiple comparisons. Performance metrics such as accuracy, sensitivity, specificity, precision, and F1-score were computed using the scikit-learn library's built-in functions, which have been extensively validated and are widely trusted in the machine learning community. These functions accept as input the ground truth labels and predicted labels, returning the desired metric values. Confusion matrices were generated similarly and visualized as heatmaps using the seaborn library, with color intensity representing the count of samples in each cell (true positive, false positive, true negative, false negative).

A critical consideration in evaluating the pipeline performance was the handling of cases where the YOLOv8 detection stage failed to identify a tooth. When a tooth was not detected (either due to poor image quality, unusual anatomy, or model error), no bounding box was generated, and consequently, no crop was created for downstream classification. These cases represent detection failures rather than classification errors. To avoid conflating detection and classification performance, such cases were systematically recorded and excluded from the denominator when computing classification metrics. However, they were included in overall system-level success rate calculations, ensuring that reported metrics accurately reflect both stages of the pipeline. This distinction is important: a model with perfect classification accuracy on successfully cropped images but poor detection recall would have limited clinical utility, whereas a model with moderate classification accuracy but excellent detection recall might still provide value. By reporting both isolated classification metrics and end-to-end pipeline metrics, the analysis provides a complete picture of system performance.

Receiver Operating Characteristic (ROC) curve analysis was conducted using SPSS version 28.0. For each CNN model configuration (three single models, three binary ensembles, one tertiary ensemble), confidence scores for each test case were imported into SPSS. The state variable (the true class label) was coded as 0 for non-C-shaped canal and 1 for C-shaped canal, while the test variable was the model's confidence score for the positive class, ranging continuously from 0 to 1. The ROC Curve procedure in SPSS then computes sensitivity and 1-specificity pairs at each unique threshold value, plots the ROC curve, and calculates the area under the curve (AUC) along with its standard error and 95% confidence interval. AUC values approaching 1.0 indicate excellent discriminative ability (the model ranks positive cases higher than negative cases in nearly all pairwise comparisons), while values near 0.5 indicate performance no better than random guessing. Separate ROC analyses were conducted for tooth #37 and tooth #47 to assess whether model performance varied between left and right mandibular second molars, which could occur if anatomical differences or image quality variations existed between the two sides.

To statistically compare AUC values between different models, the DeLong test was employed. Unlike naive z-tests that assume independence, the DeLong test accounts for the fact that multiple models are evaluated on the same set of test cases, inducing correlation between their performance estimates. The test computes a z-statistic based on the covariance structure of the pairwise comparisons, yielding a p-value that indicates whether the difference in AUC between two models is statistically significant. The DeLong test was implemented in Python using the scipy and statsmodels libraries, which provide numerically robust implementations of the algorithm. For the seven model configurations under consideration, all pairwise comparisons were conducted, resulting in 21 hypothesis tests (calculated as the binomial coefficient 7 choose 2).

Given the large number of hypothesis tests, the risk of false discoveries (Type I errors) increases substantially. If each test is evaluated at the conventional α=0.05 significance level, the expected number of false positives across 21 tests would be approximately 1.05, meaning we would incorrectly declare a difference significant roughly once even if all models truly had equal performance. To control the family-wise error rate—the probability of making at least one false discovery—a Bonferroni correction was applied. This conservative correction divides the desired overall significance level (α=0.05) by the number of tests (21), yielding a per-test significance threshold of α=0.05/21≈0.0024. Only p-values below this stringent threshold are considered statistically significant after correction. This approach ensures strong control of Type I error at the cost of reduced statistical power (increased risk of Type II errors, failing to detect true differences). In the results, both uncorrected (α=0.05) and Bonferroni-corrected (α=0.0024) p-values are reported to provide a complete view: the uncorrected results show which comparisons exhibit nominally significant differences, while the corrected results indicate which differences remain significant under stringent multiple testing correction.

This multi-faceted statistical approach—combining descriptive metrics, ROC analysis, and rigorous comparative testing—provides a thorough evaluation of model performance that meets the standards of diagnostic accuracy research as outlined in the STARD-AI guidelines. The transparency in reporting both corrected and uncorrected results, along with detailed documentation of the statistical procedures, enables readers to assess the robustness of the conclusions and facilitates meta-analyses that may aggregate results across multiple studies.
