# Supplementary material for: Automated detection of C-shaped canals in mandibular second molars from panoramic radiographs: comparing single and ensemble convolutional neural networks within a 2-stage pipeline
Source: Clin Oral Investig. 2026 Apr 23;30(5):190. doi: 10.1007/s00784-026-06868-x (PMC13102809; doi:10.1007/s00784-026-06868-x)
Supplement: Supplementary file 2 — Supplementary Material 2 (DOCX 935 KB) [file 784_2026_6868_MOESM2_ESM.docx]

**Research Article, Clinical Oral Investigations**

**Automated Detection of C-Shaped Canals in Mandibular Second Molars from Panoramic Radiographs: Comparing Single and Ensemble Convolutional Neural Networks within a 2-Stage Pipeline**

Yunus Emre Çakmak, Kürşat Er

Department of Endodontics, Faculty of Dentistry, Akdeniz University, Antalya, Türkiye

**Corresponding Author:**

Yunus Emre Çakmak, DDS

Department of Endodontics, Faculty of Dentistry, Akdeniz University, Dumlupınar Bulv., 07058 Campus, Antalya, Türkiye

**
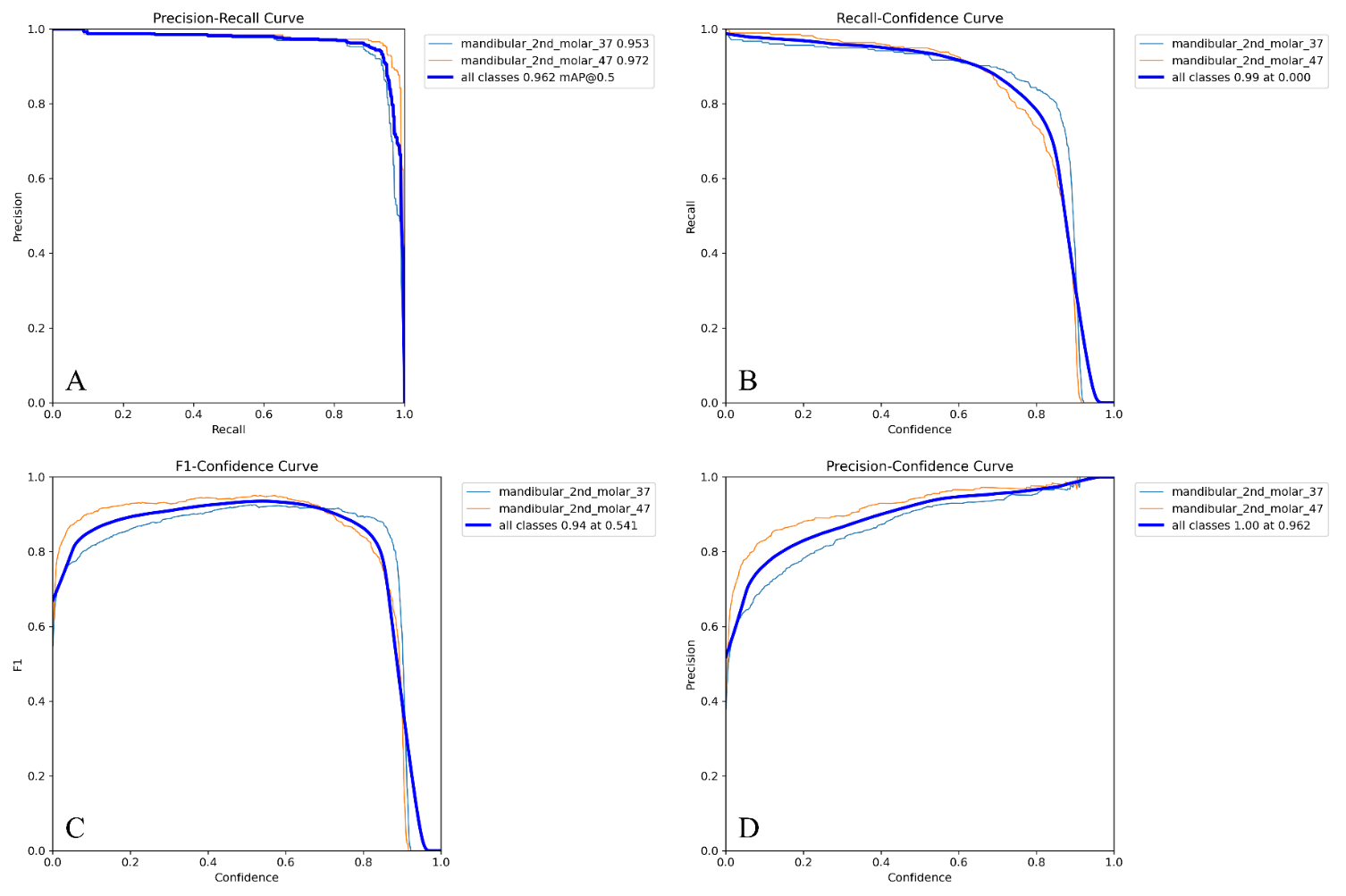
Supplementary Figure S1.** Extended training-time diagnostics for the YOLO model (computed on the validation split during training). (A) Box, classification, and DFL losses showing steady convergence without overfitting. (B) mAP@0.5 and mAP@[0.5:0.95] trajectories across epochs indicating continual performance gains. (C) Per-class precision–recall curves for tooth #37 and tooth #47, with macro-averaged PR in bold. (D) Threshold analysis (precision–confidence, recall–confidence, and F1–confidence), highlighting the operating region used in downstream experiments. Error bands denote fold-to-fold variability where applicable.


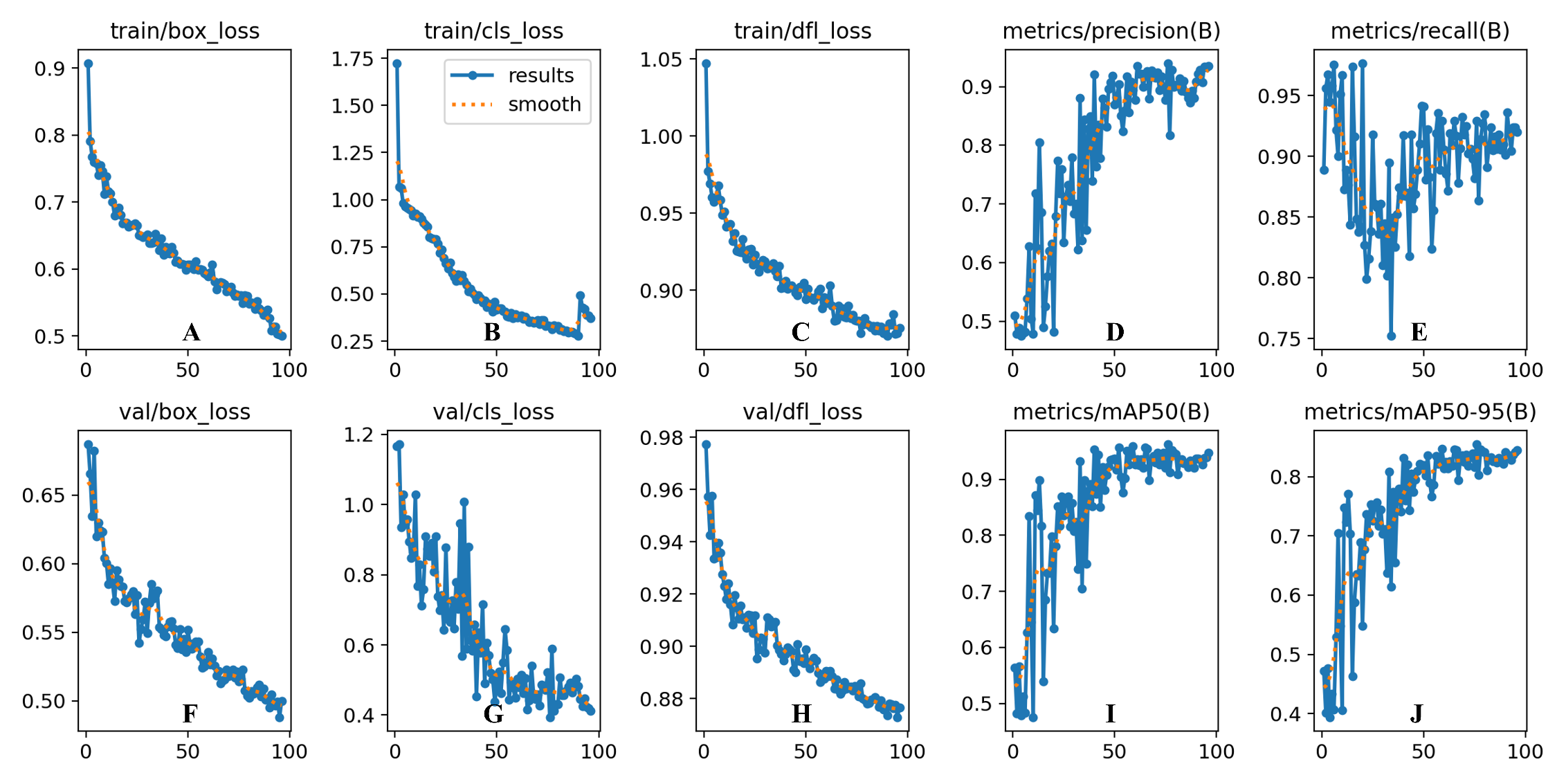


**Supplementary Figure S2.** YOLO training progress and validation metrics over 100 epochs. (A) Training box loss, (B) training classification loss (trend-smoothed), (C) training DFL loss, (D) training precision, (E) training recall. (F) Validation box loss, (G) validation classification loss, (H) validation DFL loss, (I) mAP@0.5, (J) mAP@[0.5:0.95]. All curves indicate successful convergence, with stable performance plateaus emerging around epoch ~75.


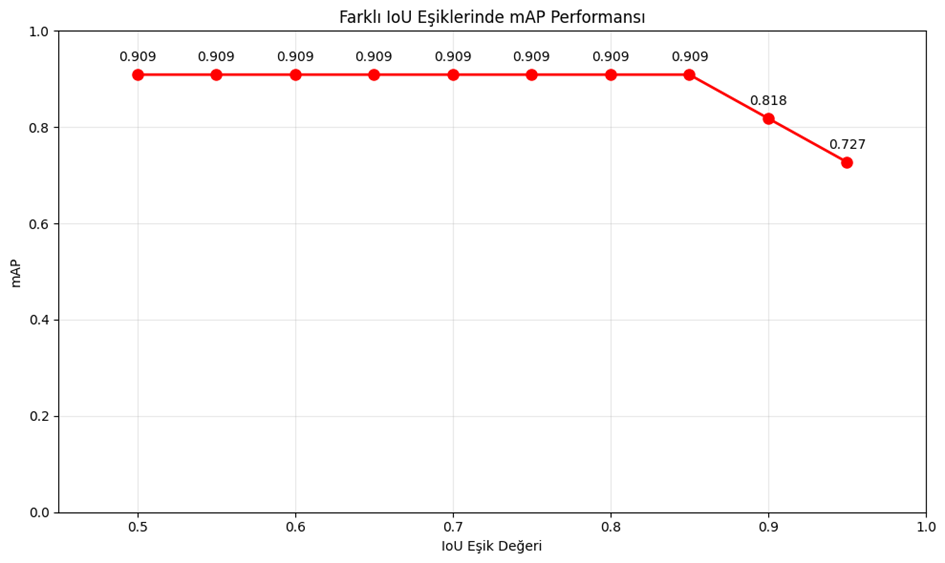


Map performance of YOLOv8 model at different IoU thresholds.

IoU thresholds

**Supplementary Figure S3.** Mean Average Precision (mAP) of the YOLOv8 model across varying IoU thresholds (0.50–0.95 in 0.05 increments), illustrating detection performance robustness over stricter localization criteria.

**Supplementary Figure S4.** Metrics of the ensemble meta-model over 100 training epochs: (A) trajectory of meta-accuracy, (B) decreasing meta-loss indicating convergence, (C) evolution of meta–F1 score reflecting the precision–recall balance, and (D) meta-sensitivity trends demonstrating the model’s ability to detect positive cases during training.**
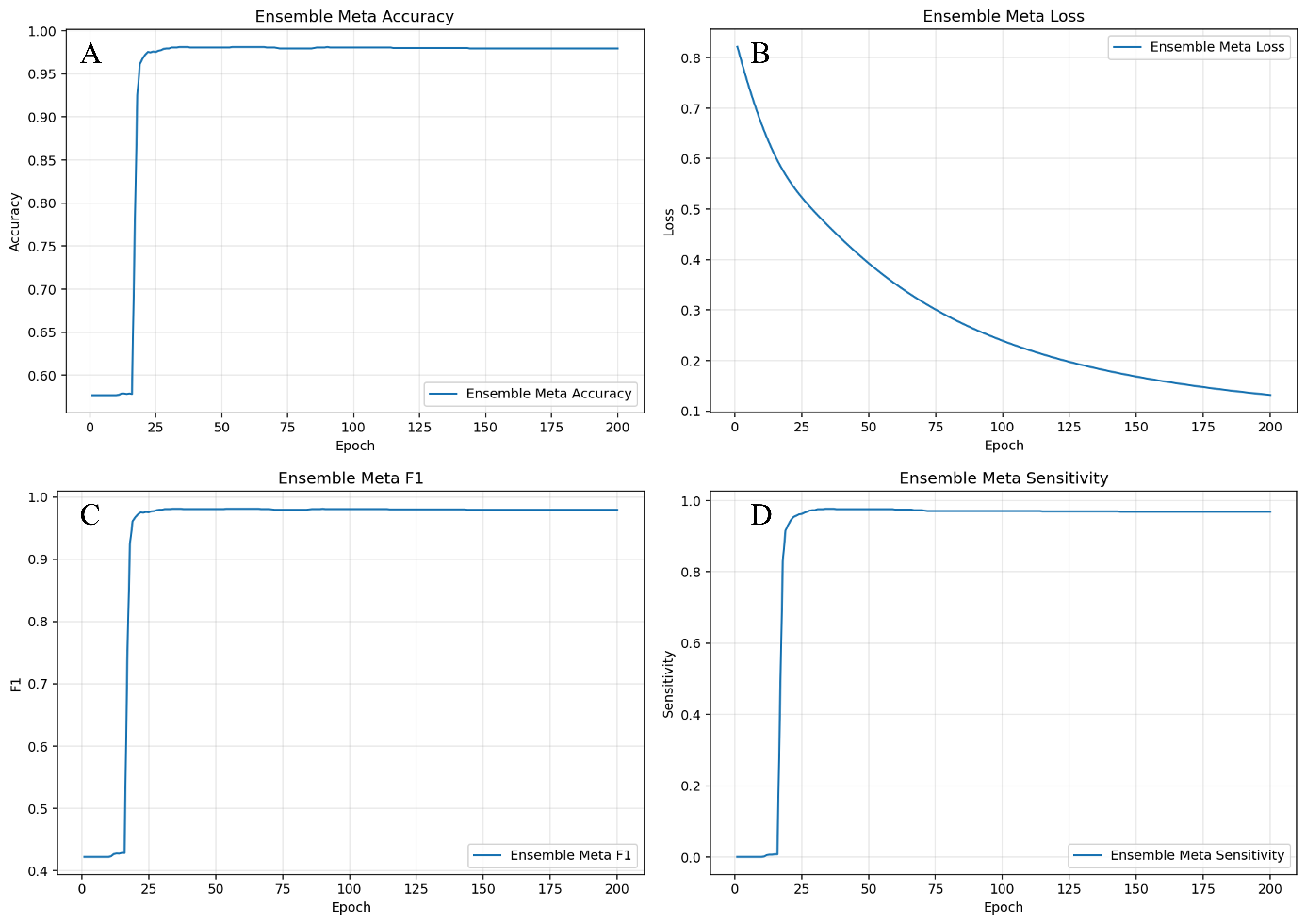
**
